# Inhibition of the mitochondrial pyruvate carrier simultaneously mitigates hyperinflammation and hyperglycemia in COVID-19

**DOI:** 10.1126/sciimmunol.adf0348

**Published:** 2023-02-23

**Authors:** Bibo Zhu, Xiaoqin Wei, Harish Narasimhan, Wei Qian, Ruixuan Zhang, In Su Cheon, Yue Wu, Chaofan Li, Russell G. Jones, Mark H. Kaplan, Robert A. Vassallo, Thomas J. Braciale, Lindsay Somerville, Jerry R. Colca, Akhilesh Pandey, Patrick E. H. Jackson, Barbara J. Mann, Connie M. Krawczyk, Jeffrey M. Sturek, Jie Sun

**Affiliations:** ^1^ Carter Immunology Center, University of Virginia, Charlottesville, VA 22908, USA.; ^2^ Division of Infectious Diseases and International Health, Department of Medicine, University of Virginia, Charlottesville, VA 22908, USA.; ^3^ Division of Pulmonary and Critical Care Medicine, Department of Medicine, Mayo Clinic, Rochester, MN 55905, USA.; ^4^ Department of Microbiology, Immunology and Cancer Biology, University of Virginia, Charlottesville, VA 22908, USA.; ^5^ Department of Metabolism and Nutritional Programming, Van Andel Institute, Grand Rapids, MI 49503, USA.; ^6^ Department of Microbiology and Immunology, Indiana University of School of Medicine, Indianapolis, IN 46202, USA.; ^7^ Department of Pathology, University of Virginia, Charlottesville, VA 22908, USA.; ^8^ Division of Pulmonary and Critical Care Medicine, Department of Medicine, University of Virginia, Charlottesville, VA 22908, USA.; ^9^ Cirius Therapeutics, Kalamazoo, MI 49007, USA.; ^10^ Department of Laboratory Medicine and Pathology, Mayo Clinic, Rochester, MN, USA.; ^11^ Manipal Academy of Higher Education, Manipal, 576104, Karnataka, India.

## Abstract

The relationship between diabetes and COVID-19 is bi-directional: while individuals with diabetes and high blood glucose (hyperglycemia) are predisposed to severe COVID-19, SARS-CoV-2 infection can also cause hyperglycemia and exacerbate underlying metabolic syndrome. Therefore, interventions capable of breaking the network of SARS-CoV-2 infection, hyperglycemia, and hyper-inflammation, all factors that drive COVID-19 pathophysiology, are urgently needed. Here, we show that genetic ablation or pharmacological inhibition of mitochondrial pyruvate carrier (MPC) attenuates severe disease following influenza or SARS-CoV-2 pneumonia. MPC inhibition using a second-generation insulin sensitizer, MSDC-0602 K (MSDC), dampened pulmonary inflammation and promoted lung recovery, while concurrently reducing blood glucose levels and hyperlipidemia following viral pneumonia in obese mice. Mechanistically, MPC inhibition enhanced mitochondrial fitness and destabilized HIF-1α, leading to dampened virus-induced inflammatory responses in both murine and human lung macrophages. We further showed that MSDC enhanced responses to nirmatrelvir (the antiviral component of Paxlovid) to provide high levels of protection against severe host disease development following SARS-CoV-2 infection and suppressed cellular inflammation in human COVID-19 lung autopsies, demonstrating its translational potential for treating severe COVID-19. Collectively, we uncover a metabolic pathway that simultaneously modulates pulmonary inflammation, tissue recovery, and host metabolic health, presenting a synergistic therapeutic strategy to treat severe COVID-19, particularly in patients with underlying metabolic disease.

## INTRODUCTION

Despite the development of many vaccines and highly successful vaccination campaigns, respiratory viruses such as influenza and SARS-CoV-2 continue to present a significant public health burden (*1*). The ever-present threat of respiratory viral infections is a result of non-sterilizing immunity induced by vaccination (*2*, *3*), as well as the constant emergence of new viral variants (*1*, *4*). Respiratory viral infections are particularly dangerous to individuals with underlying metabolic syndrome, most notably insulin resistance associated with obesity and diabetes. Indeed, hyperglycemia (high blood sugar levels) is common in hospitalized COVID-19 patients and is strongly associated with worse outcomes following SARS-CoV-2 infection (*5*, *6*). Conversely, SARS-CoV-2 promotes insulin resistance and beta cell dysfunction, inducing hyperglycemia in a significant proportion of patients without history of metabolic disease (*7*–*10*).

Immunological features of severe COVID-19 are linked to exuberant inflammation in the respiratory tract, driven by profound immune dysregulation (*11*–*14*). Thus, it is unsurprising that therapeutics targeting hyper-inflammation have shown potential in ameliorating severe COVID-19 (*15*). Indeed, the anti-inflammatory steroidal agent dexamethasone remains the most effective treatment for patients with severe COVID-19 with hypoxemia (*16*), with the restrictive therapeutic window of antiviral drugs in the early stage of infection (*17*, *18*). However, the efficacy of dexamethasone and other anti-inflammatory treatments (such as tocilizumab; anti-IL-6) are still limited (*18*). Furthermore, dexamethasone treatment often results in hyperglycemia, complicating its use in patients with underlying metabolic disease- a population exhibiting increased risk of severe COVID-19 complications (*19*, *20*). Thus, there is a clinical need to develop interventions capable of simultaneously mitigating hyper-inflammation and hyperglycemia, to address two distinct processes dysregulated in COVID-19 patients with metabolic syndrome.

Pyruvate is the end-product of glycolysis that can either be reduced to lactate in the cytosol or used as a fuel for oxidative metabolism in the mitochondria. Glycolysis is well established to have a critical role in macrophage activation and inflammatory responses (*21*). However, the contribution of pyruvate oxidation in tricarboxylic acid (TCA) cycle to macrophage function and inflammation is not well understood. Pyruvate is imported into the mitochondria via the mitochondrial pyruvate carrier (MPC), a protein known to maintain TCA cycle flux (*22*). Pyruvate fuels the TCA cycle to support anabolic metabolism and energy production. Pyruvate conversion to Acetyl-CoA supports Acetyl-CoA-dependent reactions including fatty acid oxidation and protein acetylation. Notably, MPC-mediated pyruvate metabolism has emerged as an important player in the physiological and pathophysiological processes underlying type-2 diabetes (*23*, *24*). In this report, we found that inhibition of MPC activity dampened exaggerated pulmonary inflammation and concurrently promoted host metabolic health, thereby diminishing host morbidity and mortality following influenza or SARS-CoV-2 infection in obese mice. Furthermore, MPC inhibition decreased cellular inflammation in human COVID-19 lung autopsy tissue. MPC inhibition synergized with the antiviral component of Paxlovid, nirmatrelvir, to markedly lower host mortality and lung injury in SARS-CoV-2-infected obese mice. Our results suggest that MPC inhibitor MSDC-0602 K (MSDC), a second-generation insulin sensitizer with an outstanding safety profile, has potential as a therapeutic for treating severe COVID-19, particularly in patients with underlying metabolic disease.

## RESULTS

### Genetic ablation or pharmacological inhibition of MPC function improves disease outcome following influenza or SARS-CoV-2 infection

**Fig. 1.**
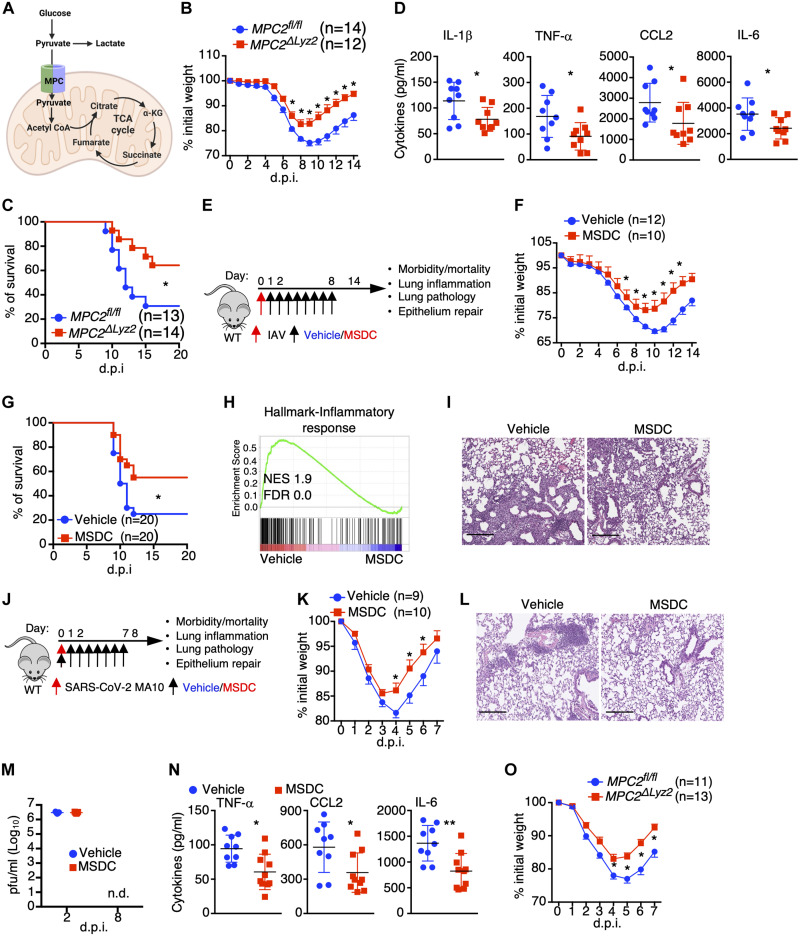
Disruption of MPC-mediated oxidative pyruvate metabolism mitigates IAV- or SARS-CoV-2-induced pathogenesis. (**A**) Diagram of pyruvate metabolic pathways. (**B** to **D**) *MPC2*^fl/fl^ and *MPC2*^ΔLyz2^ mice were infected with sub-lethal (B and D) and lethal (C) doses of IAV, respectively. Host morbidity (B) and mortality (C) were monitored. (D) BAL cytokine levels at 4 d.p.i. (n = 9). (**E**) Schematic diagram for viral infected C57BL/6 WT mice with vehicle or MSDC-0602 k (MSDC) treatment. (**F** to **I**) Mice were infected with sub-lethal (F, H, and I) and lethal (G) doses of IAV. Host morbidity (F) and mortality (G) were monitored. (H) RNA-seq analysis of lungs at 4 d.p.i. (n = 3). GSEA of inflammatory response gene set shown. (I) H&E staining of lung section (n = 4–5) at 14 d.p.i. Scale bar, 200 μm (I). (**J** to **N**) Mice were infected with SARS-CoV-2 MA10 virus. (J) Schematic diagram for viral infected C57BL/6 WT mice with vehicle or MSDC treatment. (K) Host morbidity was monitored. (L) H&E staining of lung section (n = 5) at 8 d.p.i. Scale bar, 200 μm. (M) BAL viral titers were measured at the indicated time points (n = 5). n.d., not detected. (N) BAL cytokine levels at 8 d.p.i. (n = 9–10). (**O**) *MPC2*^fl/fl^ and *MPC2*^ΔLyz2^ mice were infected with SARS-CoV-2 MA10. Host morbidity was monitored. Representative (I and L) or pooled data (B, C, D, F, G, K, N and O) from at least two independent experiments. Data are presented as means ± SEM. *, *p* < 0.05; **, *p* < 0.01; ***, *p* < 0.001. The *p* value was determined by multiple t-tests (B, F, K and O), Logrank test (C and G) or a two-tailed Student’s t-test (D, M and N).

Excessive lung macrophage activation can initiate and contribute to unrestrained inflammation through release of various pro-inflammatory mediators that lead to the recruitment of inflammatory immune cells to the lung following respiratory viral infection such as SARS-CoV-2 (*12*, *25*, *26*). Glycolysis is known to support macrophage activation and inflammation, but the importance of glucose oxidation via pyruvate translocation into the mitochondria to macrophage inflammatory responses *in vivo* is understudied (Fig. 1A). We found that mice bearing myeloid-specific deletion of the mitochondrial pyruvate transporter 2 (*MPC2*^ΔLyz2^), which forms the heteromeric MPC complex with MPC1, had decreased host morbidity and mortality following sub-lethal or lethal doses of H1N1 influenza A virus (IAV) infection (Fig. 1, B and C). *MPC2*^ΔLyz2^ mouse lungs showed comparable viral titers compared to wild type (WT) littermates but exhibited greatly diminished inflammatory gene expression in the lungs and decreased pro-inflammatory cytokine levels in bronchoalveolar lavage (BAL) fluid at 4 days post infeciton (d.p.i) (Fig. 1D; and fig. S1, A to C). *MPC2*^ΔLyz2^ mice also showed lowered accumulation of inflammatory monocytes (Ly6c^+^) and neutrophils in the lung at 4 d.p.i, which are major contributors of pulmonary immune pathology following respiratory viral infection (*27*–*29*) (fig. S1, D and E). Additionally, lung histologic tissue inflammation, disrupted alveolar areas, and total BAL protein content were all reduced in *MPC2*^ΔLyz2^ mice at 14 d.p.i (fig. S1, F and G), suggesting MPC depletion mitigates lung injury in response to IAV infection. Further, alveolar type II cell (ATII)-specific genes (such as *Abca3* and *Sftpb*) were enhanced in *MPC2*^ΔLyz2^ mice at 14 days post infection (fig. S1H), suggesting MPC depletion promotes tissue recovery. These data together suggest that genetic ablation of MPC function in myeloid cells reduces the severity of diseases following IAV pneumonia and that glucose-fueled mitochondrial metabolism likely supports pulmonary inflammation.

To determine whether pharmacological targeting of MPC could lead to attenuated viral pathology in the respiratory tract, we infected WT mice with IAV and then treated the mice with MSDC-0602 k (MSDC), which is a MPC inhibitor and also a second-generation insulin-sensitizing thiazolidinedione (TZD) that has a superior safety profile compared with first generation TZDs (Fig. 1E) (*24*). Intraperitoneal administration of MSDC from one day post IAV infection resulted in decreased host morbidity and mortality without affecting the kinetics of viral replication (Fig. 1, F and G; and fig. S1, I and J). MSDC-treated mice exhibited decreased BAL cytokine levels, diminished monocyte and neutrophil infiltration (fig. S1, K and L) and reduced lung inflammatory responses 4 d.p.i (Fig. 1H; and fig. S2, A to E). MSDC treatment also enhanced inflammation resolution, decreased lung damage and promoted ATII cell regeneration at 14 d.p.i (Fig. 1I; and fig. S2, F to H). Importantly, we did not observe a significant reduction in the magnitude of adaptive T cell response in MSDC-treated animals at 6 d.p.i, indicating its mode of action is primarily to dampen exuberant innate inflammation without interfering in antiviral adaptive immunity necessary for preventing viral dissemination and persistence (fig. S2, I to K).

Similar to IAV infection, SARS-CoV-2 infection results in profound inflammation in the lower respiratory tract (*11*). Since MSDC reduced pulmonary inflammation in IAV-infected hosts, we tested whether MSDC could dampen SARS-CoV-2-induced lung inflammation and host diseases. We challenged WT mice with a mouse-adapted SARS-CoV-2 strain MA10, which induces acute lung damage and pneumonia in mice similar to human COVID-19 (*30*), and then treated mice with vehicle or MSDC (Fig. 1J). MSDC treatment enhanced host weight recovery (Fig. 1K) and diminished pathological changes in the lungs at 8 d.p.i (Fig. 1L; and fig. S2L). This was accompanied by reduced accumulation of inflammatory monocytes and neutrophils as well as pro-inflammatory cytokine levels in the BAL, without altering viral titers (Fig. 1, M and N; and fig. S2, M and N). ATII cell loss is a prominent feature of COVID-19 (*31*), and ATII regeneration is vital for lung recovery from viral pneumonia (*32*). We found that MSDC treatment promoted ATII cell recovery in SARS-CoV-2-infected lungs at 8 d.p.i (fig. S2, O and P). Collectively, these data suggest that MSDC ameliorates pulmonary inflammation and promotes tissue recovery following SARS-CoV-2 infection.

Next, we determined whether MSDC delivered after 1 or 2 days following SARS-CoV-2 infection would still be effective. In WT mice, MSDC treatment from 1 day post-infection decreased morbidity and the efficacy diminished with treatment starting from 2 days post-infection (fig. S2, Q and R). These data suggest that the drug would be more efficacious if delivered early after viral infection, although the time “day 1” here would likely be analogous to several days after infection in human due to the delivery of a large amount of virus directly into the lung in this model. To determine whether myeloid deficiency of MPC ameliorates host morbidity, we infected *MPC2*^ΔLyz2^ mice with SARS-CoV-2 MA10 and determined host weight loss after infection. Consistent with MSDC-treated mice, myeloid MPC-deficient mice showed diminished host weight loss (Fig. 1O).

SARS-CoV-2 infection shows an age-dependent increase in disease severity (*33*, *34*). Parallel results were found in mice that became progressively more susceptible to mouse-adapted SARS-CoV-2 infection correlating with aging (*30*, *35*). We evaluated the efficacy of MSDC in aged (13–14-month-old) C57BL/6 female mice regarding SARS-CoV-2 infection (fig. S3A). In aged mice infected with SARS-CoV-2 MA10, MSDC treatment significantly reduced host mortality, with concomitant effects on weight loss (fig. S3, B and C). Additionally, MSDC diminished pathological changes in the lungs of surviving mice at 10 d.p.i (fig. S3D). These results showed that MSDC ameliorated SARS-CoV-2-induced disease in older mice.

### Murine and human lung macrophages are prominent targets of MSDC

**Fig. 2.**
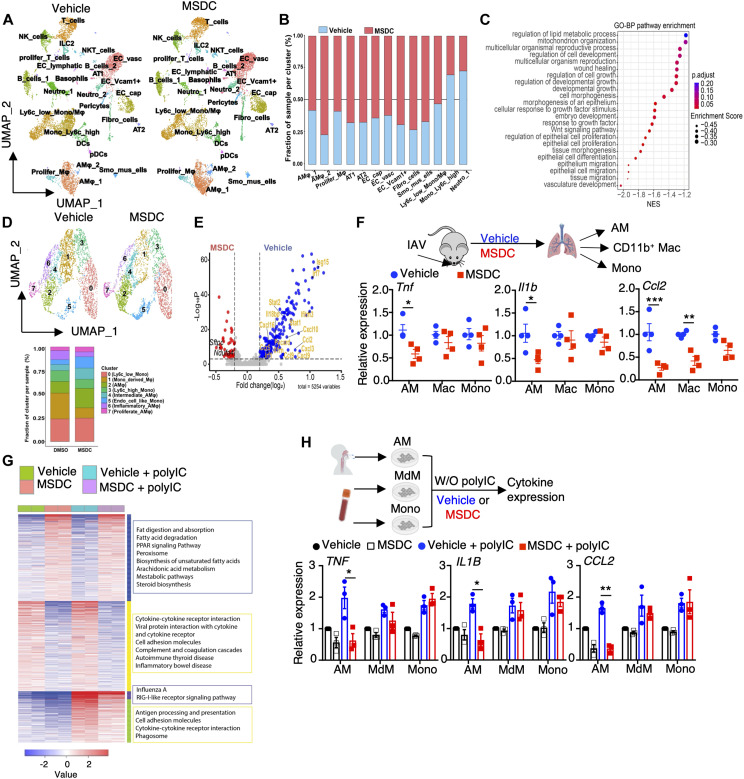
Murine and human lung macrophages are prominent target of MPC inhibition by MSDC. (**A** to **E**) scRNA-seq analysis of lungs from IAV-infected C57BL/6 WT mice with vehicle or MSDC treatment at 4 d.p.i. Lung cells were pooled from three individual mice from each group. (A) UMAP plot visualization of lung cells from vehicle- or MSDC-treated mice. (B) The relative contributions of indicated clusters by each group. (C) Dot plot showing enrichment of Gene Ontology biological processes pathways enriched in MSDC-treated lungs. The color of the dots represents the adjusted P value. Dot size represents the enrichment score. (D) UMAP showing clusters of monocytes and macrophages from (A) in vehicle- or MSDC- treated lung cells (upper panel). The percentages of each cluster in each studied subject was shown on the lower panel. (E) Volcano plot showing the differentially expressed genes in AMs (cluster 2, 4, 6 and 7) of vehicle (blue) and MSDC (red) treated mice. (**F**) The mRNA levels of *Tnf*, *Il1b* and *Ccl2* in AMs, CD11b^+^ macrophages (Mac) and monocytes (Mono) sorted from lungs at 4 d.p.i. (**G**) RNA-seq analysis of mouse AMs stimulated with or without Poly IC in the presence of vehicle or MSDC overnight *in vitro*. Heatmap of K-means clustering of differentially expressed genes and KEGG enrichment analysis. (**H**) The mRNA levels of *TNF*, *IL1B* and *CCL2* in human AMs, monocyte-derived macrophages (MdM), and monocytes (Mono) stimulated with or without Poly IC in the presence of vehicle or MSDC overnight *in vitro*. Data are presented as means ± SEM. *, *p* < 0.05; **, *p* < 0.01; ***, *p* < 0.001. The *p* value was determined by multiple t tests (F) and one-way ANOVA (H).

Single cell RNA-seq (scRNAseq) analysis revealed that lungs isolated from MSDC-treated mice exhibited relatively higher proportions of lung structural and resident immune cells including alveolar epithelial cells, endothelial cells and alveolar macrophages (AMs), but diminished infiltrating immune cells such as neutrophils, monocytes, proliferating T and plasmacytoid dendritic cells following IAV infection at 4 d.p.i (Fig. 2, A and B; and fig. S4, A and B). Consistently, MSDC-treated lungs showed enrichment of gene sets associated with wound healing, epithelial regeneration and fatty acid oxidation, while demonstrating downregulation of inflammation-associated gene sets (Fig. 2C; and fig. S4, C to E). Monocyte and macrophage-mediated inflammatory responses are considered as a major driver of respiratory viral pathogenesis (*12*, *26*). We delineated 8 subsets of monocyte and macrophage populations in the infected lungs, and MSDC treatment diminished the presence of Ly6C^+^ inflammatory monocytes, monocyte-derived macrophages (MdM) and inflammatory alveolar macrophage (AM) subsets (Fig. 2D; and fig. S4F). Notably, MSDC markedly inhibited the expression of a large number of inflammatory associated genes/sets within the total AM population, but had less prominent inhibitory effects on the inflammatory responses of monocytes or MdM (Fig. 2E; and fig. S4, G to J). RT-PCR analysis of inflammatory gene expression by sorted AMs, CD11b^+^ macrophage and monocytes further confirmed that MSDC exhibited marked anti-inflammatory effects on AMs, but with more modest effects on CD11b^+^ macrophage and monocytes at 4 d.p.i (Fig. 2F; and fig. S5A). MSDC also had modest effects in suppressing bone-marrow derived macrophages (BMDM) inflammation following Poly IC stimulation *in vitro,* compared to those of AMs (fig. S5B). RNA-seq analysis found that MSDC promoted fatty acid degradation, oxidative phosphorylation, PPAR signaling, and inhibited genes associated with interferon signaling, cytokine responses and monocyte chemotaxis in AMs with or without Poly IC treatment (Fig. 2G; and fig. S5, C to E), indicating that MSDC inhibition of MPC function profoundly altered the balance of anti-viral versus pro-inflammatory AM status. MSDC also exhibited marked anti-inflammatory effects on human AMs but showed relatively moderate immunoinhibitory effects on blood monocytes or monocyte-derived macrophages (Fig. 2H). Importantly, MSDC inhibitory effects on AM inflammatory responses was abrogated in MPC deficiency, confirming that MSDC inhibits AM inflammation via MPC (fig. S5F). Notably, while not responding to MSDC treatment, MPC-deficient-AMs showed slightly higher baseline expression of Il6 compared to MSDC-treated AMs (fig. S5F), indicating a potential off-target effect of MSDC in this setting. Together, these data indicate that lung macrophages, but not circulating monocytes, are the prominent targets of MSDC during respiratory viral infection, consistent with a recent finding that MPC function is dispensable for BMDM inflammatory responses following LPS stimulation (*36*).

### MPC inhibition by MSDC selectively reduces HIF-1α levels in lung macrophages

**Fig. 3.**
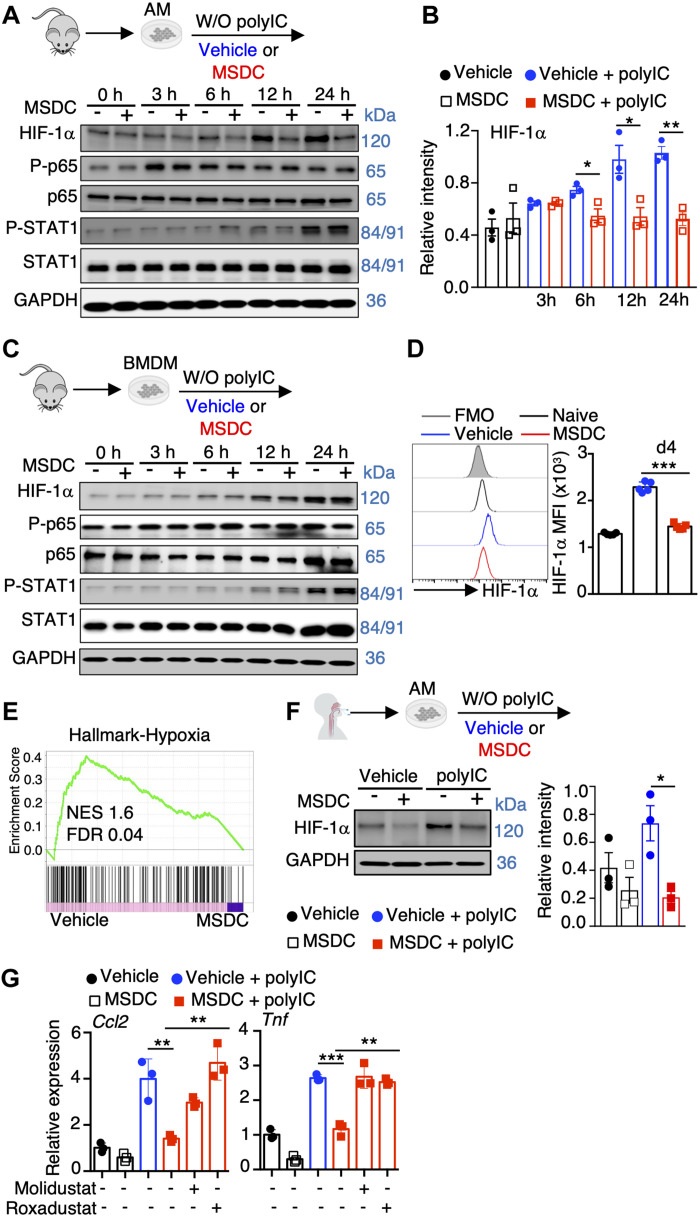
MPC inhibition dampens HIF-1α levels in lung macrophages. (**A** to **C**) Immunoblot analysis of indicated total or phosphorylated protein levels in mouse AMs (A) and BMDM (C) stimulated with or without Poly IC in the presence of vehicle or MSDC *in vitro*. (B) Quantification of HIF-1α levels in (A) of three experiments were shown. (**D**) Flow cytometry analysis of HIF-1α levels in AMs from naïve or IAV-infected wt mice treated with vehicle or MSDC at 4 d.p.i. (n = 5). (**E**) GSEA of hypoxia gene set shown for Poly IC stimulated AMs with vehicle or MSDC treatment. (**F**) Immunoblot analysis of HIF-1α in human AMs stimulated with or without Poly IC in the presence of vehicle or MSDC overnight *in vitro* (left). Quantitation on the right (n = 3 donors). (**G**) *Ccl2* and *Tnf* levels in mouse AMs under indicated conditions overnight *in vitro*. Representative immunoblots (A, C and F) were from three independent experiments. Data are presented as means ± SEM. *, *p* < 0.05. The *p* value was determined by a two-tailed Student’s t-test (B, D, and F) and one-way ANOVA (G).

To explore the molecular mechanisms by which MPC inhibition by MSDC suppressed lung macrophage inflammatory responses, we performed Western blot analysis probing HIF-1α, NF-kB and STAT-1 activation, which are known to promote macrophage inflammatory responses (*37*, *38*), following Poly IC treatment. Poly IC stimulation led to the accumulation of HIF-1α protein and higher NF-kB p65 and STAT-1 phosphorylation in AMs, and MSDC treatment inhibited HIF-1α levels but not NF-kB and STAT-1 activation (Fig. 3, A and B). In contrast, MSDC did not interfere with HIF-1α expression, p65 phosphorylation or STAT-1 activation in BMDM, consistent with its moderate effects on BMDMs (Fig. 3C). Of note, MSDC did not affect *Hif1a* mRNA levels, and MPC2 deficiency in AMs recapitulated the MSDC effects on HIF-1α protein levels (fig. S6, A and B). Additionally, MSDC treatment decreased HIF-1α levels in AMs at day 4 post IAV infection *in vivo* (Fig. 3D). Consistent with the diminished HIF-1α protein levels following MSDC treatment, MSDC treatment in Poly IC-stimulated AMs showed diminished enrichment of hypoxia-associated genes (Fig. 3E). Importantly, MSDC suppressed HIF-1α accumulation in human primary AMs following Poly IC treatment (Fig. 3F). Furthermore, *in vitro* treatment with Molidustat and Roxadustat, two HIF-1α stabilizers that promote HIF-1α accumulation in AMs, abrogated the suppressive effects of MSDC on AM inflammatory responses (Fig. 3G; and fig. S6C).

UK5099 is a well-known MPC small molecule inhibitor (*39*), although a recent manuscript has also identified off-target effects (*36*). We examined AM inflammation as well as HIF-1α expression in the presence of UK5099. MSDC and UK5099 both reduced Poly IC-induced inflammatory gene expression as well as HIF-1α level in AMs *in vitro* (fig. S6, D and E). To this end, our data from MSDC-treated MPC2-deficient AMs confirmed that the effects of MSDC on macrophage HIF-1α expression as well as inflammatory responses are largely dependent on MPC function (fig. S6B). Together, these data suggest that MSDC inhibits MPC function and promotes HIF-1α instability, thereby inhibiting lung macrophage inflammatory responses following viral stimuli.

### MPC inhibition promotes mitochondrial fitness and diminishes HIF-1α-stabilizing metabolites

**Fig. 4.**
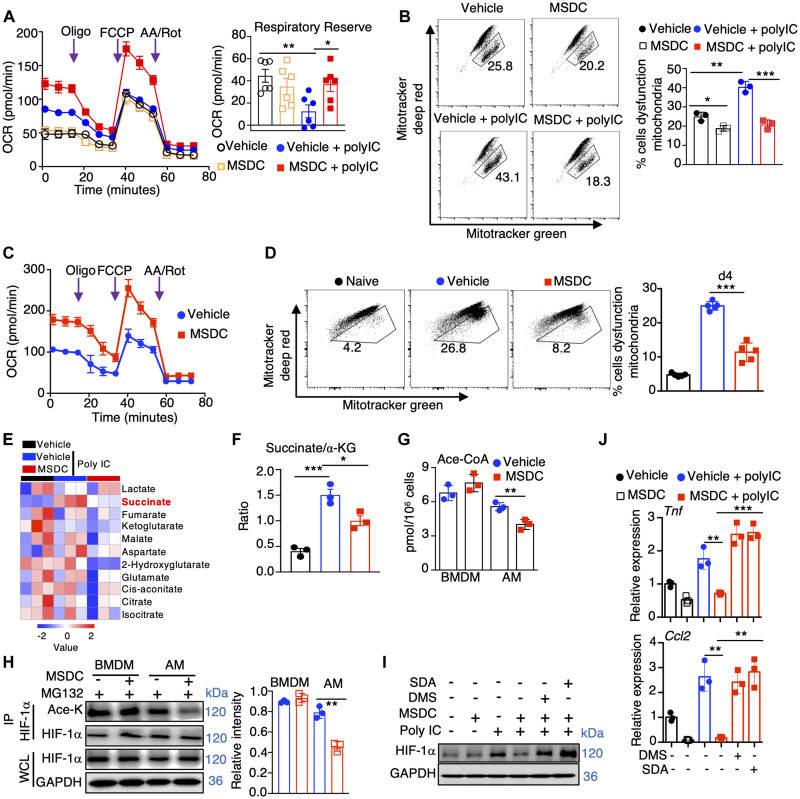
MPC inhibition promotes mitochondrial fitness and diminishes HIF-1α stabilizing metabolites in lung macrophages. (**A** and **B**) AMs were stimulated with or without Poly IC in the presence of vehicle or MSDC overnight *in vitro*. (A) OCR of AMs and quantification of respiratory reverse. (B) Flow cytometry showing mitochondrial mass by Mitotracker green versus Mitotracker deep red in AMs on the left and quantification on the right. (**C** and **D**) OCR (C) or mitochondrial mass (D) in AMs from naïve or IAV-infected wt mice treated with vehicle or MSDC at 4 d.p.i. (n = 5). (**E**) Heatmap showing TCA cycle metabolites measured in AMs (n = 3). (**F**) Succinate to Ketoglutarate (α-KG) ratios in (E). (**G**) Acetyl-CoA concentrations in BMDM and AMs treated with vehicle or MSDC overnight *in vitro*. (**H**) BMDM and AMs were stimulated with Poly IC in the presence of vehicle or MSDC overnight *in vitro*. MG132 were added 4 h before cell harvesting. Coimmunoprecipitation (IP) was performed with anti-HIF-1α antibodies followed by immunoblot analysis of HIF-1α and HIF-1α acetylation (Ace-K, with anti-ace-lysine antibodies) levels. Quantification of HIF-1α acetylation was shown. (**I** and **J**) AMs were stimulated with or without Poly IC in the presence of vehicle, MSDC, dimethyl succinate (DMS), or sodium acetate (SDA) overnight *in vitro*. (I) HIF-1α protein levels in AMs. (J) *Tnf* and *Ccl2* gene expression in AMs. Representative immunoblots (H and I) were from three independent experiments. Data are presented as means ± SEM. *, *p* < 0.05; **, *p* < 0.01; ***, *p* < 0.001. The *p* value was determined by a two-tailed Student’s t-test (A, D, F, G, and H) and one-way ANOVA (B and J).

Viral stimuli have been demonstrated to inhibit macrophage mitochondrial metabolism, facilitating macrophage-mediated inflammatory responses (*34*, *40*). MSDC treatment enhanced maximal oxygen consumption rate (OCR) and respiratory reserve in AMs, but not BMDMs, following stimulation with Poly IC (Fig. 4A; and fig. S7A). Consistent with these observations, fewer depolarized mitochondria were seen in MSDC-treated AMs but not BMDM populations (Fig. 4B; and fig. S7B). Additionally, MSDC treatment increased respiratory reserve and mitochondrial fitness in AMs at 4 days post IAV infection *in vivo* (Fig. 4, C and D). These data suggest that MSDC promotes mitochondrial respiration in lung macrophages. In support of this conclusion, AMs, but not lung monocytes, exhibited enrichment of genes associated with mitochondrial oxidative phosphorylation *in vivo* during infection after MSDC treatment (fig. S7, C and D). MSDC treatment caused enhanced extracellular acidification rate (ECAR) potentially due to the increased lactic acid accumulation after the blockade of pyruvate translocation into mitochondria both in AMs and BMDMs (fig. S7, E and F) (*36*, *41*).

Pyruvate is oxidized in the TCA cycle following its translocation into mitochondria (*22*). We therefore measured TCA metabolites following *in vitro*. Poly IC stimulation in the presence or absence of MSDC. Notably, Poly IC greatly promoted the accumulation of succinate in AMs and BMDMs, which was diminished in the presence of MSDC in AMs but not BMDMs (Fig. 4E; and fig. S7G). High succinate/α-KG ratio is an indication of reduced complex II activity of the electron transport train (ETC). MSDC treatment also reduced the succinate/α-KG ratio in AMs but not BMDMs, consistent with improved mitochondrial respiration (Fig. 4F; and fig. S7H). HIF-1α protein can be stabilized by both succinate and acetylation (*42*, *43*). We measured Acetyl-CoA levels and found that MSDC reduced Acetyl-CoA accumulation in AMs, but not BMDMs (Fig. 4G). Reduced Acetyl-CoA levels could affect gene expression by reducing histone acetylation, however, MSDC treatment did not markedly suppress the acetylation of total H3 or H3K27 (fig. S7I). Rather, diminished Acetyl-CoA in AMs was associated with decreased HIF-1α acetylation (Fig. 4H) (*42*, *43*). We next sought to determine whether exogenous succinate or Acetyl-CoA could promote AM HIF-1α levels and promote their inflammatory responses following MSDC treatment. We treated Poly IC- and/or MSDC-stimulated AMs with sodium acetate (SDA) or dimethyl succinate (DMS) to boost intracellular Acetyl-CoA and succinate, respectively. Exogenous SDA or DMS treatment promoted HIF-1α accumulation in AMs and enhanced *Tnf* and *Ccl2* expression even in the presence of MSDC (Fig. 4, I and J), indicating that diminished succinate and/or Acetyl-CoA levels likely contribute to decreased HIF-1α following MSDC treatment in AMs.

Previous studies in T cells and other cell types have found that inhibition of pyruvate translocation led to increased glutamine and lipid incorporation into the mitochondria (*44*). Next, we sought to investigate whether increased mitochondrial respiration in AMs following disruption of pyruvate metabolism by MSDC could be due to the increased utilization of fatty acid or glutamine oxidation. We performed Seahorse assays in AMs in the presence of glutaminase inhibitor BPTES and/or carnitine palmitoyltransferase-1 inhibitor etomoxir in the context of MPC inhibition *in vitro*. MSDC treatment increased oxidative phosphorylation (OXPHOS) and mitochondrial fitness compared to vehicle treatment; however, single and particularly the combined treatment of BPTES and/or etomoxir inhibited OXPHOS and mitochondrial fitness compared to MSDC alone treated cells (fig. S8). These data suggest that the increased mitochondrial fitness in AMs following MPC inhibition is likely due to the compensatory effects of glutamate and/or fatty acid oxidation.

Previously, we have shown that genetic HIF-1α deficiency in AMs or inhibition of HIF-1α stability by LW6 administration *in vivo* diminished lung inflammation following IAV infection (*38*). Similarly, we found that LW6 treatment decreased host morbidity and inflammation following SARS-CoV-2 infection (fig. S9, A to F), suggesting that increased HIF-1α expression is a primary driver of pulmonary inflammatory responses. HIF-1α is known to promote IL-1β production in macrophages, and IL-1β release is a major contributor of pulmonary inflammation during COVID-19 (*42*, *45*, *46*). Next, we sought to determine whether inhibition of HIF-1α-dependent IL-1β contributed to decreased macrophage inflammation by MSDC. We treated WT or *Il1b*-deficient AMs with MSDC or LW6 in the presence or absence of Poly IC. MSDC or LW6 diminished proinflammatory cytokine expression in both WT or *Il1b*-deficient AMs following Poly IC stimulation (fig. S9G), suggesting that the decreased inflammatory capacity of AMs by MSDC or HIF-1α inhibitor treatment is independent of IL-1β *in vitro.* Consistently, blockade of IL-1β did not significantly decrease host morbidity as seen in MSDC- or LW6-treated mice *in vivo* (fig. S9H). Therefore, we conclude that the diminished macrophage inflammation following MSDC treatment is unlikely to be due to diminished IL-1β production after treatment. Together, these data suggest that MPC inhibition by MSDC improves mitochondrial metabolism and diminishes the accumulation of metabolites capable of stabilizing HIF-1α, thereby suppressing the inflammatory responses of lung macrophages following respiratory viral infection.

### MSDC promotes metabolic health and concurrently suppresses pulmonary hyper inflammation

**Fig. 5.**
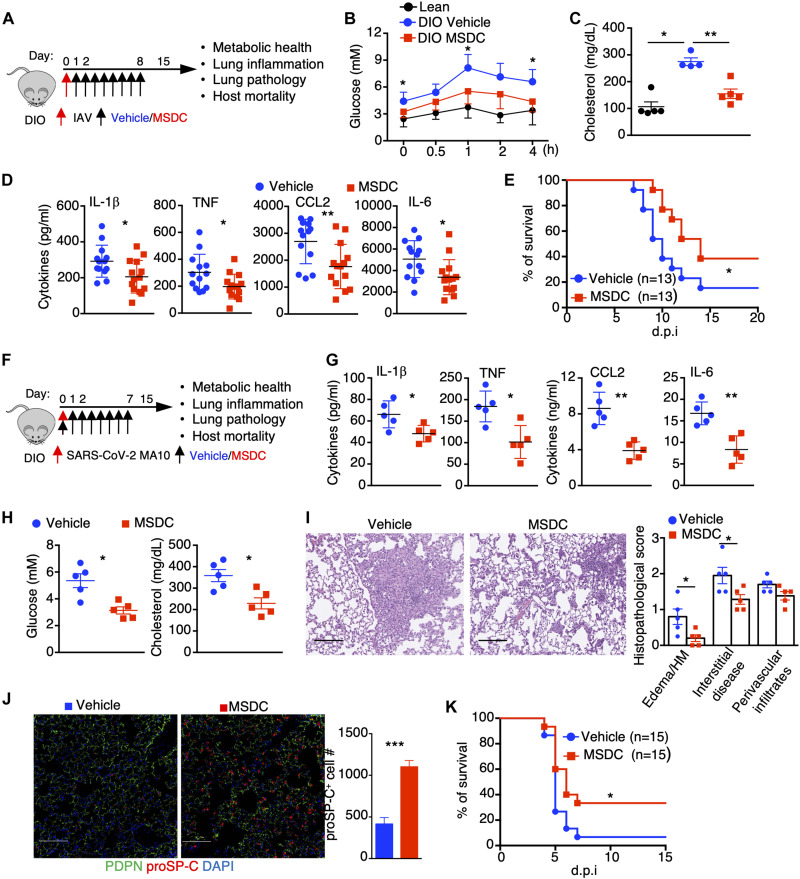
MSDC treatment simultaneously promotes host metabolic health, dampens pulmonary inflammation and enhances tissue recovery. (**A**) Schematic diagram for treatment of IAV-infected DIO mice. (**B** to **E**) DIO mice were infected with sub-lethal (B to D) or lethal (E) doses of IAV and treated with vehicle or MSDC. (B) Blood glucose concentration measured by an i.p. glucose tolerance test at 5 d.p.i. (n = 9–10). (C) Total cholesterol concentrations in the blood at 5 d.p.i. (n = 4–5). (D) BAL cytokine levels at 5 d.p.i. (n = 13–14). (E) Host mortality was monitored. (**F** to **K**) DIO mice were infected with SARS-CoV-2 MA10 virus and treated with vehicle or MSDC. (F) Schematic diagram. (G) BAL cytokine levels at 5 d.p.i. (n = 5). (H) Blood glucose and total cholesterol levels were measured at 5 d.p.i. (n = 5). (I) H&E staining of lung section (n = 5) and quantification of pathological lesions at 5 d.p.i. Scale bar, 200 μm. HM, hyaline membranes. (J) Fluorescence microscopy images of PDPN, proSP-C and DAPI staining in fixed lung tissues at 5 d.p.i (n = 5). Scale bar, 50 μm. Quantification of proSP-C^+^ cell number was performed using at least 10 random fields (10x) of alveolar space per mouse lung. (K) Host mortality was monitored. Representative (I and J) or pooled data (B, D, E and K) from at least two independent experiments. Data are presented as means ± SEM. *, *p* < 0.05; **, *p* < 0.01. The *p* value was determined by Logrank test (E and K), one-way ANOVA (B), or a two-tailed Student’s t-test (C, D and G to J).

Respiratory virus infections are particularly dangerous to people who have underlying metabolic syndromes, most notably insulin resistance with obesity and diabetes (*5*, *47*). This paradigm is observed in models where, compared to lean mice, obese mice also showed increased morbidity and mortality following IAV or SARS-CoV-2 infection (*48*–*50*). Since MSDC has been found safe and effective in lowering glycemia and liver steatosis (*24*), and dampening IAV-induced inflammation in lean host (Fig. 1), we tested the therapeutic efficacy of MSDC in ameliorating IAV pneumonia in obese mouse models (Fig. 5A). High fat diet (HFD)-induced obese (DIO) mice showed higher levels of blood glucose and total cholesterol at 5 d.p.i compared to lean mice, while MSDC administration improved glucose tolerance and lowered total cholesterol in the blood compared to vehicle-treated DIO mice (Fig. 5, B and C). Furthermore, MSDC-treated mice had decreased cytokine levels in the BAL and a marked reduction in expression of multiple pro-inflammatory genes in the lung (Fig. 5D; and fig. S10, A and B), but similar viral titers compared to vehicle-treated mice at 5 d.p.i (fig. S10C). MSDC treatment also promoted lung inflammation resolution and recovery as evidenced by less disrupted lung tissue areas, diminished BAL protein levels and enhanced expression of ATII-specific genes at the recovery stage following MSDC treatment (15 d.p.i.) (fig. S10, D to F). Similar phenotypes of MSDC treatment were observed in IAV-infected leptin receptor mutant (db/db) mice (fig. S11), indicating that MSDC can simultaneously promote metabolic heath and dampen lung inflammation following IAV infection in both genetically- or diet-induced obesity models. Consistent with these observations, MSDC treatment reduced host mortality following IAV infection in DIO mice (Fig. 5E).

Emerging evidence has suggested that obesity predisposes hosts to severe COVID-19 following SARS-CoV-2 infection (*5*, *6*, *47*). Our data also indicated that DIO mice showed increased host mortality than lean mice following SARS-CoV-2 infection (fig. S12A). To this end, we examined the therapeutic potential of MSDC in mitigating severe diseases following SARS-CoV-2 MA10 infection in obese mice (Fig. 5F). MSDC-treated DIO mice had lower levels of pro-inflammatory cytokines, which was accompanied with decreased numbers of inflammatory monocytes and neutrophils in the BAL at 5 d.p.i (Fig. 5G; and fig. S12, B and C), suggesting MPC inhibition dampened SARS-CoV-2-induced pulmonary inflammation. MSDC administration also decreased glucose and cholesterol levels in the blood at 5 d.p.i (Fig. 5H), indicating that MSDC ameliorated metabolic conditions in obese mice after SARS-CoV-2 infection. Additionally, MSDC treatment lowered lung inflammatory cytokine expression, BAL protein levels and reduced disrupted alveolar areas by lung histopathological analysis without affecting viral gene expression (Fig. 5I; and fig. S12, D and E). MSDC-treated lungs showed increased ATII gene expression and elevated levels of ATII cell presence at 5 d.p.i (Fig. 5J; and fig. S12F), which suggest that MSDC can potently enhance lung recovery and regeneration following SARS-CoV-2 infection in obese hosts. Consequently, SARS-CoV-2-induced lethality was partially abrogated following MSDC treatment at 6 h or 1 day post-infection, while most of the vehicle-treated DIO mice had succumbed to SARS-CoV-2 infection (Fig. 5K; and fig. S12G). These data suggest that MPC inhibition by MSDC diminishes pulmonary hyperinflammation while concurrently promoting metabolic health following respiratory viral pneumonia in hosts with underlying metabolic conditions.

### MSDC diminishes cellular inflammation in COVID-19 lung autopsies and enhances response to anti-viral therapy

**Fig. 6.**
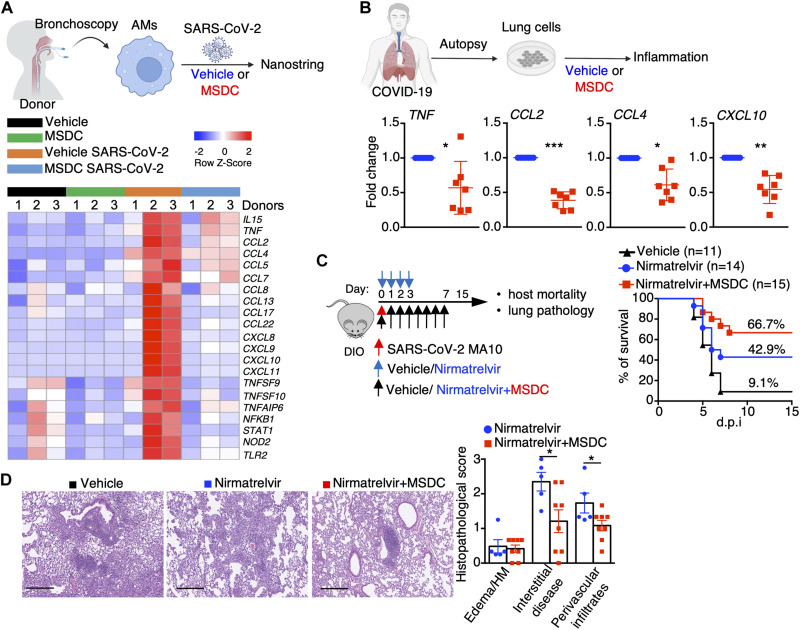
MSDC diminishes COVID-19-associated lung inflammation and exhibits synergy with anti-viral therapy. (**A**) Human AMs from BAL of non-infectious donors were infected with or without SARS-CoV-2 in the presence of vehicle or MSDC (n = 3 donors) for 48 h *in vitro*. Nanostring analysis of inflammatory genes in AMs. (**B**) Cytokine expression in lung cells from COVID-19 patient autopsies following vehicle or MSDC treatment overnight *ex vivo* (n = 7 subjects). (**C** and **D**) DIO mice were infected with SARS-CoV-2 MA10 virus and treated with vehicle, Nirmatrelvir, or Nirmatrelvir plus MSDC. (C) Host mortality was monitored and survival rate is shown. (D) H&E staining of lung section (n = 5) and quantification of pathological lesions at 21 d.p.i. Scale bar, 200 μm. HM, hyaline membranes. Representative (D) or pooled data (B and C) from at least two independent experiments. Data are presented as means ± SEM. *, *p* < 0.05; **, *p* < 0.01; ***, *p* < 0.001. The *p* value was determined by paired t test (B), Logrank test (C), and a two-tailed Student’s t-test (D).

We next sought to further explore the translational potential of MSDC in treating COVID-19, particularly in patients with metabolic conditions. Infection of macrophages by SARS-CoV-2 has emerged as an important contributor to COVID-19 associated inflammation (*45*, *51*). We thus examined whether MSDC could diminish human lung macrophage inflammatory responses following SARS-CoV-2 infection. SARS-CoV-2 infection caused markedly elevated inflammatory gene expression in AMs isolated from two of three healthy donors, but MSDC treatment markedly inhibited a large number of inflammatory genes upregulated following SARS-CoV-2 infection in AMs (Fig. 6A; and fig. S13, A and B). We next examined whether MSDC could dampen lung inflammatory responses in COVID-19 patients to determine its potential as a treatment for severe COVID-19. To this end, we incubated total lung cells from seven deceased COVID-19 patients with or without MSDC, and determined *TNF, CCL2, CCL4* and *CXCL10* expression in the lung (Fig. 6B; and fig. S13C). Notably, MSDC treatment inhibited the expression of these inflammatory genes in cells from the lungs of COVID-19 patients (Fig. 6B; and fig. S13C), demonstrating the potential of MSDC in treating exuberant pulmonary inflammation in severe COVID-19.

Finally, since many patients at-risk for severe disease from COVID-19 are now likely to be treated with anti-viral drugs such as Paxlovid, we examined whether MSDC could work with anti-viral therapies to provide an added level of protection against severe COVID-19, in this setting. We treated SARS-CoV-2 MA10-infected DIO mice with nirmatrelvir (the anti-viral component of Paxlovid) at 6 h post-infection in the absence or presence of MSDC (Fig. 6C). Notably, MSDC plus nirmatrelvir treatment protected the majority of mice from death following SARS-CoV-2 infection in obese mice, whereas nirmatrelvir alone appeared less efficacious (Fig. 6C). Furthermore, lungs from mice treated with MSDC plus nirmatrelvir showed less tissue inflammation and alveolar disruption compared to mice treated with nirmatrelvir alone (Fig. 6D), indicating that MSDC in combination with antiviral therapy can mitigate severe COVID-19 in at-risk hosts. Taken together, these data support a potential role for MSDC in the treatment of COVID-19 in patients with metabolic conditions, particularly when combined with anti-viral therapy such as nirmatrelvir.

## DISCUSSION

Lung macrophage populations are heterogeneous immune sentinel cells, critical for antiviral innate immunity and tissue recovery following respiratory viral infections (*52*). Conversely, macrophage-derived inflammatory and/or injurious mediators also contribute to excessive pulmonary inflammation and collateral tissue injury following viral infections (*12*, *38*, *53*). Indeed, the aberrant activation of monocytes or resident macrophages is considered a key driver of virus-induced inflammation in severe COVID-19 (*12*, *51*, *54*). However, specific pathways and/or mediators that can be targeted to dampen exuberant macrophage-mediated lung inflammation without compromising beneficial antiviral immunity remain largely unknown. Here, we showed that mitochondrial pyruvate translocation is selectively required for detrimental lung macrophage-mediated inflammatory responses following viral infections including SARS-CoV-2. Moreover, this pathway can be safely targeted to improve outcomes following severe viral pneumonia using the second-generation TZD MSDC (fig. S14).

AMs are among the first immune cells responding to viral infections. The inflammatory mediators produced by AMs not only directly contribute to pulmonary inflammation, but could also indirectly augment the overall levels of inflammation by recruiting inflammatory immune cells such as monocytes and neutrophils following infection (*25*, *38*, *55*). Thus, the inhibition of AM inflammatory responses by MSDC has the potential to directly and/or indirectly dampen the pathogenic inflammation after respiratory viral infection. Additionally, we did observe modest inhibitory effects of MSDC on MdM and monocyte inflammatory responses *in vivo*. Thus, MSDC may function to inhibit the inflammatory responses of both resident and recruited macrophages to mitigate severe disease development post viral infection.

While glycolysis is well-established to be involved in inflammatory responses for both monocytes and macrophages (*56*), MPC-dependent pyruvate oxidation appears to be preferentially required for pulmonary macrophage-mediated inflammation by stabilizing HIF-1α. This distinct feature of MPC-dependent inflammatory activity in lung macrophages may be particularly meaningful and exploited to dampen pulmonary inflammation. Unlike corticosteroid treatment such as dexamethasone that induces a systemic anti-inflammatory state often resulting in secondary infections and complications (*19*, *57*), the immunosuppressive effect of the MPC inhibitor may be limited to the respiratory tract. Interestingly, inhibition of pyruvate flux into mitochondria can lead to compensatory metabolic reactions including increased fatty acid and/or glutamine oxidation in T and other cell types (*44*), which is concordant with our transcriptomic analysis and increased mitochondrial respiration. Notably, fatty acid and glutamine oxidative metabolism is known to promote pro-recovery M2-like macrophages (*58*), consistent with the observation that MSDC treatment enhanced lung recovery and regeneration following viral clearance. Thus, MSDC may also serve as a pro-reparative therapeutic in the clinic by augmenting lung tissue recovery (such as ATII cell replenishment) following COVID-19 lung injury.

Growing evidence indicates that SARS-CoV-2 induces mitochondrial dysfunction in immune cells. Acute SARS-CoV-2 infection resulted in rapid mitochondrial dysfunction in both CD4 and CD8 T cells, which compromised "T cell" functionality contributing to suppressed "T cell" immune responses to viral infection (*59*). Patients with SARS-CoV-2 infection displayed depolarized mitochondria and abnormal mitochondrial ultrastructure in monocytes, which was correlated with enhanced inflammatory responses (*60*). Recently, targeted transcriptome analysis also revealed impairment of mitochondrial OXPHOS and anti-oxidant gene expression in autopsy samples, which was associated with enhanced HIF-1α stabilization (*61*, *62*). Thus, means that can promote mitochondrial metabolic fitness may be promising for developing novel therapeutic avenue for COVID-19. In line with this notion, our study also showed compromised mitochondrial respiration and increased HIF-1α expression in AMs after viral infection *in vivo* (*40*), and the inhibition of pyruvate metabolism by MSDC enhanced mitochondrial OXPHOS and fitness, which was associated with the reduction of proinflammatory cytokines.

Obesity and/or diabetes greatly increases the risk of severe disease following IAV or SARS-CoV-2 infections (*5*, *63*). Correction of insulin resistance and hyper-glycemia using insulin sensitizer drugs has been suggested for the management of diabetic patients with COVID-19 (*5*). Metformin, the most prescribed anti-diabetic drug, has been suggested as a repurposed drug for COVID-19 due to its anti-inflammatory properties (*64*, *65*). Nevertheless, metformin failed to provide significant clinical benefits in randomized placebo-controlled clinical trials (*66*, *67*), and the efficacy of other anti-diabetic drugs such as Glucagon-like peptide-1 receptor agonists (GLP1-RAs) also remains controversial (*68*). Therefore, interventions capable of circumventing the deadly cycle of viral infection, hyperglycemia and hyper-inflammation are critical for improving treatment of severe COVID-19 patients with history of metabolic disease. First generation TZDs, including Pioglitazone and Rosiglitazone, are effective in treating type 2 diabetes (*69*), but induce considerable side effects, resulting in their restriction and removal from the market in many countries. MSDC is a second-generation insulin sensitizer, maintaining all the pharmacological benefits of first generation TZDs, without the potential for edema and exhibiting an outstanding safety profile as per a recent multicenter, double-blinded, randomized controlled trial (*24*). Importantly, our data also shows that MSDC treatment is effective when combined with a current standard-of-care antiviral therapy.

We have shown that MSDC can simultaneously dampen hyperglycemia and hyperinflammation in obese hosts. Of note, hyperglycemia per se predisposes the host to more severe disease development following viral infection including SARS-CoV-2. Therefore, we have not assessed the relative contribution of hyperglycemia versus macrophage inflammatory activities in driving the severity of respiratory viral infection during obesity. Future experiments utilizing MPC myeloid conditional deficient mice under the obesity setting would be ideal to study this question. Additionally, our data showed that inhibition of glutaminase and/or carnitine palmitoyltransferase-1 disrupted elevated mitochondrial metabolism in response to MSDC treatment, suggesting that the increased fitness and respiration of mitochondria after MPC inhibition is likely due to the increased glutamine and/or fatty acid oxidation. However, such a model can not be definitively established without analyzing the metabolic flux using specific isotope tracers. Unfortunately, such tracing experiments remain impractical due to requiring a large number of primary tissue macrophages. Nevertheless, our data have uncovered a metabolic pathway that concurrently modulates macrophage inflammation, lung recovery and host metabolic health, and suggest a potentially viable therapeutic agent that may be combined with existing anti-viral agents to treat severe COVID-19 in patients with underlying metabolic disease.

## MATERIALS AND METHODS

### Study design

The aim of this study was to determine the therapeutic potential of mitochondrial pyruvate carrier inhibitor MSDC-0602 K (MSDC) in treating severe viral infection including SARS-CoV-2 infection in normal and obese hosts. We examined the efficacy of MSDC in dampening host diseases and promoting metabolic health following influenza virus and mouse-adapted SARS-CoV-2 MA10 virus infection in lean and obese mouse models. ScRNA-seq, bulk RNA-seq, metabolic analysis and flow cytometry were used to uncover the cellular, molecular and metabolic mechanisms by which MSDC regulates lung macrophage inflammatory process following influenza or SARS-CoV-2 infection. Lastly, we tested the potential roles of MSDC in regulating SARS-CoV-2-induced inflammation in humans by culturing SARS-CoV-2-infected primary human lung macrophages and human COVID-19 lung autopsy samples *in vitro* with or without MSDC. Viral infections in mice were ended upon mouse sacrifice at indicated days after infection. In general, experiments were conducted in replicates and the number of mice used in the studies were included in figure legends.

### Ethics and biosafety

The study involving human participants was reviewed and approved by Mayo Clinic Institutional Review Boards (IRB# 19–012187). All animal experiments were performed in animal housing facilities at the Mayo Clinic (Rochester, MN) or the University of Virginia (UVA, Charlottesville, VA). Sex matched and age matched adult mice of both sexes unless otherwise specified were used in the experiments. The animal experiments were approved by the the Mayo Clinic or UVA Institutional Animal Care and Use Committees (IACUC). All work with SARS-CoV-2 infection was approved for use under ABSL3/BSL3 conditions, and was performed with approved standard operating procedures and safety conditions by UVA Institutional Review Board.

### Mouse and infection

WT C57BL/6 (Cat# 000664), Lyz2-cre (Cat# 004781), and *Mpc2^fl/fl^* (Cat# 032118) mice were purchased from the Jackson Laboratory and bred in house. *Mpc2^ΔLyz2^* mice were generated by crossing *Mpc2^fl/fl^* mice with Lyz2-cre mice. High fat diet-induced obese (DIO) male mice on C57BL/6 background (with 60% Kcal fat chow for 20 weeks) (Cat# 380050) and age/sex-matched control mice (Cat# 380056) were purchased from the Jackson Laboratory and bred in house for another 2 weeks with 60% Kcal fat chow (Research Diets, Cat# D12492) or normal chow before experiments. All mice housed in a specific pathogen-free environment. For host morbidity experiments following regular dose of influenza A virus (IAV) infection, influenza A/PR8/34 strain was diluted in FBS-free DMEM media on ice and inoculated in anesthetized mice through intranasal route. For host mortality experiments following high dose (2.5 folds of the sublethal dose, lethal) of IAV infection, the outcome was determined based on the humane endpoint (more than 30% weight loss or moribund) or deaths before humane sacrifice as described before (*38*). For SARS-CoV-2 MA10 infection, mice were infected with 10^5^ PFU (for 9–12 weeks old WT C57BL/6 mice), 8x10^4^ PFU (for aged C57BL/6 mice) or 10^4^ PFU (for 26–28 weeks old DIO mice) of MA10 intranasally under anesthesia. Body weight was monitored daily for virus infected mice.

### MPC inhibitor MSDC-0602 k treatment *in vivo*

MSDC-0602 k (MSDC) was kindly provided by Cirius Therapeutics, which was developed for nonalcoholic steatohepatitis (NASH). MSDC was dissolved in DMSO. For treatment of IAV-infected WT C57BL/6 lean mice or DIO mice, mice were administered by intraperitoneal (for C57BL/6 lean mice) or oral gavage (for DIO mice) daily with either 5% DMSO as vehicle or 30 mg/kg MSDC in 200 μl PBS from 1 d.p.i to 8 d.p.i. unless otherwise specified. For treatment of SARS-CoV-2 MA10-infected WT C57BL/6 mice, aged C57BL/6 mice or DIO mice, mice were administered by intraperitoneal (for C57BL/6 mice or aged C57BL/6 mice) or oral gavage (for DIO mice) daily with either 5% DMSO as vehicle or 30 mg/kg MSDC in 200 μl PBS from 6 hours post infection to 7 d.p.i. unless otherwise specified. Since DIO mice have greatly enhanced morbidity and mortality after infection, we reasoned that for DIO mice, i.p. injection of 200ul liquid daily is a huge burden, therefore, we chose to use oral gavage. For treatment of SARS-CoV-2 MA10-infected DIO mice with Nirmatrelvir, Nirmatrelvir (PF-07321332) was purchased from MedChemExpress (Cat# HY-138687). The compounds were dissolved in DMSO and formulated as 40 mg/ml in corn oil containing 10% DMSO. DIO mice were administered by oral gavage with either 10% DMSO as vehicle, 300 mg/kg Nirmatrelvir or 300 mg/kg Nirmatrelvir plus 30 mg/kg MSDC. The treatment of Nirmatrelvir or MSDC was initiated at 6 h post MA10 infection, and continued twice daily for a total of 3 days for Nirmatrelvir or continued once daily for a total of 7 days for MSDC, respectively. For treatment of SARS-CoV-2 MA10-infected WT C57BL/6 mice, mice were administered by intraperitoneal with 400 μg IgG (BioXcell, Cat# BE0091) or Anti-IL-1β (BioXcell, Cat# BE0246) antibodies at day 1 and day 3 post infection. Mice were monitored for body weight change. At indicated time points, a subset of mice was euthanized and lung or BAL samples were collected for inflammation and titre analysis. Another subset of mice were euthanized and half of each lung lobe was taken for histopathology and were fixed in 10% phosphate buffered formalin before paraffin embedding and sectioning, and half of lung lobe was taken for further analysis.

### Human AM culture and treatment *in vitro*

For human AMs, we selected donors without a history of immunosuppression and chemo or radiotherapies, and are free of inflammation or pulmonary infection. The study was reviewed and approved by the Institutional Review Board (IRB# 19–012187) at Mayo Clinic. All participants provided written informed consent prior to sample collection and subsequent analysis.

Human AMs were obtained from Broncho-alveolar lavage (BAL) of adult donors undergoing flexible bronchoscopy as described before (*38*). About 100 to 200 ml of saline were instilled in 20-ml aliquots until 60 ml of lavage fluid was obtained. The specimen was placed on ice and immediately hand carried to laboratory for cell isolation. AMs were purified by adherence for 2 h in complete medium (RPMI-1640, 10% FBS, 1% Pen/Strep/glutamate) at 37 °C and 5% CO_2_. The non-adherent cells were washed off with warm PBS. The remaining adherent cells were cultured in complete medium supplemented with 50 ng/ml recombinant human GM-CSF (Biolegend, Cat# 572903) and M-CSF (Biolegend, Cat# 574804). For Poly IC treatment, AMs were pre-treated with DMSO (vehicle) or MSDC (10 μM) for two hours, then, cells were stimulated with or without Poly IC (5 μg/mL) for 24 hours and were analyzed by quantitative RT-PCR.

For SARS-CoV-2 infection, AMs were pre-treated with DMSO (vehicle) or MSDC (10 μM) for two hours. Subsequently, cells were washed with cold PBS and challenged with or without 1 MOI of SARS-CoV-2 virus for one hour, and then cultured in the presence of DMSO or MSDC (10 μM) for 48 hours. Cells were analyzed by quantitative RT-PCR or Nanostring.

### Human lung tissue specimens

Lung autopsy samples from 7 individuals who died from COVID-19 were obtained from Mayo Clinic Department of Pathology. Informed consent was obtained from relatives of study participants. Lung tissue specimens were obtained within 24 h of autopsy and immediately processed for single cell suspension. For lung cells treatment *ex vivo*, the cells were treated with DMSO (vehicle) or MSDC (10 μM) in complete medium supplemented with 50 ng/ml recombinant human GM-CSF and M-CSF for 24 h. Cells were analyzed by quantitative RT-PCR.

### Glucose tolerance test

Glucose tolerance test was performed as described before (*70*). Briefly, DIO mice were weighed and fasted overnight at 4 d.p.i. Then the mice were injected intraperitoneally with 1 g/kg D-glucose in 0.9% NaCl immediately after collecting blood from tail vein (T = 0) at 5 d.p.i. Subsequently, the blood was obtained at 0.5, 1, 2 and 4 h post injection from tail vein. Blood glucose was measured with 2 μl of serum from each blood sample at indicated time points by colorimetric glucose assay kit (Abcam, Cat# ab65333) according to manufacturer’s instructions.

### Total cholesterol detection

Total cholesterol concentrations in each blood sample were measured with colorimetric cholesterol assay kit (Abcam, Cat# ab65390) according to manufacturer’s instructions. Intra-assay C.V.’s was 0.8%, 0.8% and 0.7% at 85, 178 and 340 mg/dL respectively.

### Mouse AM culture and treatment *in vitro*

Mouse AMs were obtained from BAL as described previously (*26*). Briefly, alveolar lavages were pooled from BAL washes (PBS with 2 mM EDTA). AMs were purified by adherence for 2 h in complete medium (RPMI-1640, 10% FBS, 1% Pen/Strep/glutamate) at 37°C and 5% CO_2_. The non-adherent cells were washed off with warm PBS. The remaining adherent cells were cultured in complete medium supplemented with 10 ng/ml recombinant murine GM-CSF (Biolegend, Cat# 576304). For Poly IC treatment, AMs were pre-treated with DMSO (vehicle), MSDC (10 μM), UK5099 (20 μM, Selleckchem), BPTES (10 μM, TargetMol), or Etomoxir (20 μM, Sigma-Aldrich) for two hours, then, cells were stimulated with or without Poly IC (5 μg/mL) for 24 hours and were analyzed by quantitative RT-PCR. For HIF-1α stabilizers treatment, AMs were pre-treated with DMSO (vehicle) or MSDC (10 μM) with or without the HIF-1α stabilizers, 100 μM Molidustata (MedChemExpress, Cat# HY-12654) or 100 μM Roxadustat (MedChemExpress, Cat# HY-13426) for two hours. Subsequently, cells were stimulated with or without Poly IC (5 μg/mL) for 24 hours. For boost intracellular Acetyl-CoA and succinate assay, AMs were pre-treated with DMSO (vehicle) or MSDC (10 μM) with or without 20 mM sodium acetate (Sigma, Cat# S8625) or 20 mM dimethyl succinate (Sigma, Cat# W239607) for two hours. Subsequently, cells were stimulated with or without Poly IC (5 μg/mL) for 24 hours. Cells were analyzed by quantitative RT-PCR or Western blot.

### Acetyl-Coenzyme A measurement

Mouse AMs or BMDMs were pre-treated with DMSO (vehicle) or MSDC (10 μM) for two hours, then, cells were stimulated with Poly IC (5 μg/mL) for 24 hours. The concentration of acetyl-CoA is quantified by BioVision’s PicoProbe Acetyl CoA Assay Kit (Cat# MAK039), according to the protocols provided by the manufacturer.

### Bulk RNA sequencing

Total RNA from lungs of IAV-infected mice and *in vitro* cultured AMs were used for bulk RNA sequencing. After quality control, high quality (Agilent Bioanalyzer RIN >7.0) total RNA was used to generate the RNA sequencing library. cDNA synthesis, end-repair, A-base addition, and ligation of the Illumina indexed adapters were performed according to the TruSeq RNA Sample Prep Kit v2 (Illumina, San Diego, CA). The concentration and size distribution of the completed libraries was determined using an Agilent Bioanalyzer DNA 1000 chip (Santa Clara, CA) and Qubit fluorometry (Invitrogen, Carlsbad, CA). Paired-end libraries were sequenced on an Illumina HiSeq 4000 following Illumina’s standard protocol using the Illumina cBot and HiSeq 3000/4000 PE Cluster Kit. Base-calling was performed using Illumina’s RTA software (version 2.5.2). Paired-end RNA-seq reads were aligned to the mouse reference genome (GRCm38/mm10) using RNA-seq spliced read mapper Tophat2 (v2.2.1). Pre- and post-alignment quality controls, gene level raw read count and normalized read count (i.e. FPKM) were performed using RSeQC package (v2.3.6) with NCBI mouse RefSeq gene model. Differential expression for each gene between various groups specified in the text was identified on basis of the results of DESeq2 Wald tests. For visualization, data were logarithmic transformed, and genes that exhibited log_2_ fold change values >2 and log_10_ P > 25 between compared groups were highlighted. For functional analysis, gene set enrichment analysis (GSEA) (*71*) was applied to identify enriched gene sets from MSigDB, using a weighted enrichment statistic and a log_2_ ratio metric for ranking genes. The Bulk RNA sequencing was conducted once using multiple biological samples per group (as indicated in figures).

### Single-cell RNA sequencing

C57BL/6 WT mice were infected with ~200 PFU IAV and treated with vehicle or MSDC for 3 days. Lung cells were pooled from three individual mice from each group at 4 d.p.i, and subjected to scRNA-seq analysis. Single-cell libraries were prepared using the Chromium Single Cell 5’ Reagent Kit (10x Genomics) following manufacturer’s instruction. Paired-end sequencing was performed using an DNBSEQ-G400 in rapid-run mode. scRNA-seq data were aligned and quantified using 10x Genomics Cell Ranger Software Suite. Subsequently, the doublet cells cells were removed by the package “scDblFinder”. Remaining cells were analyzed using “Seurat” (version 4.1.1). The following criteria were applied for quality control: gene number > 200, UMI count >1,000 and mitochondrial gene percentage < 5%. The workflow included normalization, dimension reduction, and clustering, as well as identification of marker genes for clusters and differentially expressed genes. Gene set enrichment analysis (GSEA) (*71*) analysis is based on the results of FindAllMarkers with the package of clusterProfiler (*72*).

### Metabolic analysis

Real-time oxygen consumption rate (OCR) and extracellular acidification rate (ECAR) of AMs or BMDM were measured using a Seahorse XFp Analyzers (Seahorse Bioscience) (*38*). 1 × 10^5^ a.m. or BMDM were seeded into each well of Seahorse XFp Cell Culture Miniplates, and pre-treated with DMSO (vehicle) or MSDC (10 μM) for two hours, then, cells were stimulated with or without Poly IC (5 μg/mL) for overnight at 37°C and 5% CO_2_. On the following day, the cells were washed twice and incubated at 37°C for 1 hr. in the absence of CO_2_ in unbuffered assay medium (pH = 7.4, Agilent Technologies) with 10 mM glucose for mitochondrial stress test (or without glucose for glycolytic stress test). OCR and ECAR were measured under basal conditions and after the addition of the following compounds: 1 μM oligomycin, 1.5 μM FCCP (carbonyl cyanide-4-(trifluoromethoxy) phenylhydrazone), 0.5 μM rotenone +0.5 μM antimycin, 10 mM glucose, and 50 mM 2-DG (2-deoxy-D-glucose) (all compounds obtained from Sigma) as indicated. Data were analyzed with Wave Desktop software version 2.6 (Agilent Technologies).

### Measurement of mitochondrial mass

1 × 10^5^ a.m. or BMDM were seeded into 24 well plates and pre-treated with DMSO (vehicle) or MSDC (10 μM) for two hours, then, cells were stimulated with or without Poly IC (5 μg/mL) for overnight at 37°C and 5% CO_2_. On the following day, the cells were washed and rinsed and incubated with Mitotracker deep red (Invitrogen, Cat# M22426) and Mitotracker green (Invitrogen, Cat# M7514) at 50 nM for 30 min at 37°C. Then, cells were washed twice with PBS and lifted off the plates for flow cytometry.

### Metabolite analysis

For TCA-analytes testing as described before (*73*), 2 million of mouse AMs or BMDMs were treated with DMSO (vehicle) or MSDC (10 μM) in the presence or absence of Poly IC *in vitro*. The cell pellets were lyzed in 50 μl of acidified 1X PBS after spiking in 15 μl of internal solution containing U-13C labeled analytes. The proteins were removed by adding 260 μl of chilled methanol and acetonitrile solution to the sample mixture. After drying the supernatant in the speed vac, the sample was derivatized with ethoxime and then with MtBSTFA +1% tBDMCS (N-Methyl-N-(t-Butyldimethylsilyl)-Trifluoroacetamide +1% t-Butyldimethylchlorosilane) before it was analyzed on an Agilent 5975C GC/MS (gas chromatography/mass spectrometry) under electron impact and single ion monitoring conditions. Concentrations of lactic acid (m/z 261.2), fummaric acid (m/z 287.1), succinic acid (m/z 289.1), oxaloacetic acid (m/z 346.2), ketoglutaric acid (m/z 360.2), malic acid (m/z 419.3), aspartic acid (m/z 418.2), 2-hydroxyglutaratic acid (m/z 433.2), cis aconitic acid (m/z 459.3), citric acid (m/z 591.4), and isocitric acid (m/z 591.4), glutamic acid (m/z 432.4) were measured against a 7-point calibration curves that underwent the same derivatization.

### Flow cytometry analysis

Fluorescence-conjugated flow cytometry antibodies (Abs) were purchased from Biolegend and BD Biosciences. Cell suspensions were stained with the appropriate antibody cocktail in flow cytometry buffer at 4°C for 30 min. The cell populations were defined based on following cell surface markers as described previously (*38*): AMs (CD11c^+^ Siglec F^+^ CD11b^low^ CD64^+^ MerTK^+^), Neutrophils (CD11b^+^ Ly6G^+^), total CD11b^+^ Monocyte/Macrophage population (Ly6G^−^ Siglec F^−^ CD11b^+^), inflammatory Monocytes (Ly6G^−^ Siglec F^−^ CD11b^+^ Ly6C^+^), interstitial macrophages (Siglec F^−^ CD11b^+^ CD64^+^ MerTK^+^), NP_366_ tetramer^+^ cells (CD8^+^ NP_366_-tet^+^), PA_224_ tetramer^+^ cells (CD8^+^ PA_224_-tet^+^). The information for those Abs as follows: anti-SiglecF-BV421 (BD Biosciences, clone E50–2440, Cat# 562681), anti-CD11b-PerCP-Cy5.5 (Biolegend, clone M1/70, Cat# 101228), anti-CD11c-BV510 (Biolegend, clone N418, Cat# 117338), anti-Ly6G-PE-Cy7 (Biolegend, clone 1A8, Cat# 127618), anti-Ly6C-BV711 (Biolegend, clone HK1.4, Cat# 128037), anti-CD64-PE (Biolegend, clone X54–5/7.1, Cat# 139304), anti-MerTK-APC (Biolegend, clone 2B10C42, Cat# 151508), anti-CD4-BV510 (Biolegend, clone RM4–5, Cat# 100559), anti-CD8-PerCP-Cy5.5 (Biolegend, clone YTS156.7.7, Cat# 126610), Influenza NP_366_ Tetramer (NIH Tetramer Facility, Cat# H-2D(b) ASNENMETM), Influenza PA_224_ Tetramer (NIH Tetramer Facility, Cat# H-2D(b) SSLENFRAYV). The dilution of surface staining Abs was 1:200. Staining samples were collected on FACS Attune or FACS Attune NXT flow cytometer (Life technologies) and analyzed using Flow Jo software (Tree Star).

For intracellular staining, cell suspensions were stained for surface marker at 4°C for 30 min. Cells were washed twice with FACS buffer (PBS, 2 mM EDTA, 2% FBS, 0.09% Sodium Azide), prior to fixation and permeabilization with the Foxp3 transcription factor staining buffer set (eBioscience) for 1 h at RT in the dark. Cells were washed twice with perm wash buffer (eBioscience), and stained with Abs against HIF-1α (clone 241812, R&D Systems, Cat# IC1935P) and control immunoglobulin (Biolegend) for at least 30 min at RT, and washed twice with perm wash. Samples were processed with flow cytometer.

### Statistical analysis

Data are mean ± SEM of values from individual mice (*in vivo* experiments). Unpaired two-tailed Student’s t-test (two group comparison), one-way ANOVA (multiple group comparison), Multiple t-tests (weight loss) or Logrank test (survival study) were used to determine statistical significance by GraphPad Prism software. We consider *P* values <0.05 as significant. *, p < 0.05; **, p < 0.01.

## Supplementary Materials

### Other Supplementary Material for this manuscript includes the following:

Data file S1

MDAR Reproducibility Checklist

## Notes

View/request a protocol for this paper from *Bio-protocol*.
