# Supplementary material for: Inhibition of the mitochondrial pyruvate carrier simultaneously mitigates hyperinflammation and hyperglycemia in COVID-19
Source: Sci Immunol. 2023 Feb 23:eadf0348. doi: 10.1126/sciimmunol.adf0348 (PMC9972900; doi:10.1126/sciimmunol.adf0348)
Supplement: Supplementary file 1 — Materials and Methods Figs. S1 to S14 Table S1 [file sciimmunol.adf0348_sm.pdf]

Science Immunology
AAAS

Supplementary Materials for

# Inhibition of the mitochondrial pyruvate carrier simultaneously mitigates hyperinflammation and hyperglycemia in COVID-19

Bibo Zhu *et al.*

Corresponding author: Jie Sun, js6re@virginia.edu

*Sci. Immunol.* **8**, eadf0348 (2023)
DOI: 10.1126/sciimmunol.adf0348

**The PDF file includes:**

Materials and Methods
Figs. S1 to S14
Table S1

**Other Supplementary Material for this manuscript includes the following:**

Data file S1
MDAR Reproducibility Checklist

**Supplementary Materials and Methods:**

**Generation of mouse adapted SRAS-CoV-2 MA10 stocks**

Mouse adapted SARS-CoV-2 (MA10 variant) (BEI Resources, Cat # NR-55329) was generated by passaging on Vero E6 cells at 37°C (passage 2, SARS-CoV-2 P2) (*30*). SARS-CoV-2 MA10 P2 was used for all *in vivo* experiments. Working stock titers were determined using plaque assay. Vero E6 cells were cultured in Dulbecco's modified Eagle's medium (DMEM, Gibco) with the addition of 2% Fetal Clone II serum (Hyclone) and 1% Pen/Strep/glutamate. Serial dilutions were added to the cells. The plate was incubated at 37°C, 5% $CO_2$ for 1 hour, shaking the plates every 15 minutes. After incubation, monolayers were overlayed with media containing 1.2% Avicel PH-101 (Sigma Aldrich, St. Louis, MO) and incubated at 37°C, 5% $CO_2$. After 72 hours, the overlay was removed, wells were fixed with 10% formaldehyde, and stained with 0.1% crystal violet to visualize plaques. Plaques were counted, and PFUs were calculated according to the following equation: Average # of plaques/dilution factor × volume diluted virus added to the well.

**HIF-1α inhibitor treatment *in vivo***

HIF-1α inhibitor LW6 was purchased from TargetMol (Cat# T3494), and was dissolved in DMSO. For treatment of SARS-CoV-2 MA10-infected WT C57BL/6 mice, mice were administered by intraperitoneal daily with either DMSO as vehicle or 30 mg/kg LW6 in 200 µl PBS from 6 hours post infection to 7 d.p.i. Mice were monitored for body weight change. At indicated time points, a subset of mice was euthanized and lung or BAL samples were collected for further analysis as described above.

**Human monocytes and monocyte-derived macrophages culture and treatment *in vitro***

Human monocytes cells from healthy donors were isolated from whole blood. Before applying the MACS® systems, 10 mL whole blood were diluted with 10 mLPBS, and then added 10 mL Ficoll-Paque (Cytiva) to the bottom of the tube. Peripheral blood mononuclear cells (PBMC) were isolated by density gradient centrifugation (400g, 40 min, room temperature). Monocytes were isolated from PBMC by MACS® CD14 MicroBeads (Miltenyi Biotec, Cat# 130-050-201) according to the manufacturer's protocol. Monocytes were cultured in RPMI-1640 supplemented with 10% FBS and 1% Pen/Strep/glutamate at 37℃ and 5% $CO_2$. To obtain monocytes derived

macrophages (MdM), monocytes were incubated with 50 ng/mL of M-CSF for 7 days with 1/3 of medium renewed at day 3. For Poly IC treatment, monocytes or MdM were pre-treated with DMSO (vehicle) or MSDC (10 µM) for two hours, then, cells were stimulated with or without Poly IC (5µg/mL) for 24 hours and were analyzed by quantitative RT-PCR.

**Mouse BAL Fluid**

BAL fluid was obtained by flushing the airway three times with a single inoculum of 600 µL sterile PBS via a trachea incision. Cells in BAL fluid was spun down for flow cytometry analysis and supernatants were collected for the determination of cytokines/chemokines levels, protein concentration and viral titers.

**ELISA analysis of BAL cytokines**

50 µl of each BAL sample was analyzed with the ELISA using commercially available kits for mouse IL-1β (Biolegend, Cat# 432604), TNF-α (Biolegend, Cat# 430904), IL-6 (Biolegend, Cat# 431304) and CCL2 (Biolegend, Cat# 432701) following the manufacturer's protocol. The VERSAmax microplate reader (Molecular Devices) was used for colorimetric quantification and analysis at 450 nm wavelength.

**BCA protein assay**

BCA protein assay kit was obtained from Thermo Scientific (Cat# 23225). 2 µl of each BAL sample was used. VERSAmax microplate reader (Molecular Devices) was used for colorimetric quantification and analysis at 570nm wavelength.

**Virus titer measurement**

For IAV, the viral titers in the BAL were measured by endpoint dilution assay and expressed them as tissue culture infectious dose 50 per ($TCID_{50}$) as described before (38). Briefly, Madin-Darby canine kidney cells (MDCK, The American Type Culture Collection) were grown in 96-well plates and incubated with 10-fold dilutions of BAL sample from IAV-infected mice with different treatments in serum-free DMEM medium. After a 3-day incubation at 37 °C in a humidified atmosphere of 5% $CO_2$, the supernatants were collected and mixed with a half-volume of 0.5% chicken red blood cells (Rockland, Cat# R401-0100). After 30 min incubation at

room temperature (RT), the agglutination pattern was analyzed and the $TCID_{50}$ values were calculated.

For SARS-CoV-2 MA10, the viral titer in the BAL were determined using plaque assay. Vero E6 cells were cultured in DMEM with the addition of 2% Fetal Clone II serum (Hyclone) and 1% Pen/Strep/glutamate. Serial dilutions were added to the cells. The plate was incubated at 37°C and 5% $CO_2$ for 1 hour, shaking the plates every 15 minutes. After incubation, monolayers were overlayed with media containing 1.2% Avicel PH-101 and incubated at 37 °C and 5% $CO_2$. After 72 hours, the overlay was removed, wells were fixed with 10% formaldehyde, and stained with 0.1% crystal violet to visualize plaques. Plaques were counted, and PFUs were calculated according to the following equation: Average # of plaques/dilution factor × volume diluted virus added to the well.

**Mouse bone marrow derived macrophage (BMDM) culture and treatment *in vitro***

BMDM were isolated from bone marrow of C57BL/6 mice. The bone marrow cavities were repeatedly flushed with RPMI-1640 containing 10% FBS. After red cells were removed, the remained cells were cultured in RPMI-1640 supplemented with 50 ng/mL of M-CSF (Biolegend, Cat# 576404), 10% FBS and 1% Pen/Strep/glutamate at 37 °C and 5% $CO_2$ for 7 days with medium renewed at day 4. For Poly IC treatment, BMDMs were pre-treated with DMSO (vehicle) or MSDC (10 µM) for two hours, then, cells were stimulated with or without Poly IC (5µg/mL) for 24 hours and were analyzed by quantitative RT-PCR or western blot.

**Lung immunofluorescence**

Paraffin-embedded lung tissue sections were deparaffinized in xylene and rehydrated. Heat-induced antigen retrieval was performed using Agilent Dako target retrieval solution (pH 9; Cat# S2367), followed by permeabilization with 0.1% Triton-X with 0.05% Tween-20 for 1 hour at room temperature. Sections were next stained with the following primary antibodies: Hamster anti-PDPN (1:750; Abcam, Cat# ab11936) or Rabbit anti-proSP-C (1:500; Millipore, Cat# AB3786) diluted in Agilent Dako Background reducing antibody diluent (Cat# S3022) overnight at 4 °C. Subsequently, samples were washed and incubated with fluorescent secondary antibodies: Goat anti-hamster AlexaFluor488 (1:400; Invitrogen, Cat# A-21110) and Donkey anti-rabbit DyLight 649 (1:125; Biolegend, Cat# 406406), in the dark for 2 hours at room

temperature. After washes, the samples were stained with DAPI (1:1000, ThermoFisher Scientific) for 3 minutes and mounted using ProLong Antifade mountant (ThermoFisher Scientific, Cat# P36930). Images were acquired using the Olympus BX63 fluorescent microscope and pseudocolours were assigned for visualization. For each lung section, images were taken in at least 10-12 random alveolar areas. All images were further processed using the ImageJ Fiji software.

**Lung cell suspension preparation**

lungs of treated mice were cut into small pieces, and digested with Collagenase Type 2 (Worthington Biochemical) for 30 min at 37 °C. Cells were further passed through 70 μm cell strainer and washed with flow cytometry buffer. After red blood cell lysis, cells were centrifuged and re-suspended in appropriate buffer for flow cytometry analysis or stimulation *in vitro*.

**RNA extraction**

For lung homogenate from IAV- or SARS-CoV-2-infected mice, human AMs with SARS-CoV-2 infection, lung cells from COVID-19 patient autopsy, RNA was extracted using the TRIzol/chloroform method (ThermoFisher, Cat# 15596018). For *in vitro* cultured mouse AMs and BMDM and human AMs, MdM, and monocytes, RNA was isolated using Total RNA purification kit and treated with DNase I (Sigma, Cat# RTN350) according to the manufacturer's instructions.

**Quantitative RT-PCR**

Random primers and Moloney murine leukemia virus (MMLV) reverse transcriptase (Invitrogen, Cat# 28025021) were used to synthesize first-strand cDNAs from equivalent amounts of RNA from each sample. Quantitative PCR with reverse transcription (RT-qPCR) was performed with Fast SYBR Green PCR Master Mix (Applied Biosystems, Cat# 4385618). RT-qPCR was conducted in duplicates in QuantStudio3 (Applied Bioscience). Data were generated with the comparative threshold cycle (Delta CT) method by normalizing to hypoxanthine phosphoribosyltransferase (HPRT). Sequences of primers used in the studies are listed in Table S1.

**Immunoprecipitation and Immunoblots**

Cells were lyzed in RIPA buffer (Sigma, CAT# R0278) with a protease inhibitor cocktail (Sigma, CAT# P8340) and 4x sample loading buffer (containing SDS and 2-mercaptoethanol). Proteins were separated by electrophoresis through 4% -12% SDS-PAGE. For Immunoprecipitation assay, BMDM and AMs were stimulated with Poly IC in the presence of vehicle or MSDC overnight *in vitro*. 0.5 μM MG132 were added 4 h before cell harvesting. Cells were lysed in RIPA buffer for 30 minutes on ice. A portion of the lysate was taken as the input, and the remaining lysate was immunoprecipitated using anti-HIF-1α and subjected to Western blotting. The antibodies used in immunoblots and co- immunoprecipitations were as follows: HIF-1α (Cell Signaling Technology, Cat# 14179S), NF-κB p65 (Cell Signaling Technology, Cat# 8242S), Phospho-NF-κB p65 (Cell Signaling Technology, Cat# 3033S), STAT1 (Cell Signaling Technology, Cat# 14994S), Phospho-STAT1 (Cell Signaling Technology, Cat# 9167S), acetyl lysine antibody (Abcam, Cat# ab21623), MPC2 (Cell Signaling Technology, Cat# 46141S), Histone H3 (Cell Signaling Technology, Cat# 4499S), acetyl-Histone H3 (Millipore, Cat# 06-599), Acetyl-Histone H3 (Lys27) (Cell Signaling Technology, Cat# 8173S) and GAPDH (Cell Signaling Technology, Cat# 97166S).

**Lung histopathology**

Mice were perfused with PBS (10 mL) via the right ventricle following euthanasia. Paraformaldehyde (PF, 10%) was then gently instilled into the lung and left inflated for one minute before excising and moving lobe to 10% PF for 48 hours followed by transfer to ethanol (70%). Samples were shipped to Mayo Clinic Histology Core Lab (Scottsdale, AZ) where they were embedded in paraffin and 5 μm sections were cut for Hematoxylin and eosin stain and immunofluorescence studies. The slides were scanned through the Aperio whole slide scanning system (Leica) and exported to image files. Lung histopathology was blindly scored using four random fields per tissue as previously described (*35*). Tissues were evaluated for the presence of edema or hyaline membranes using distribution-based ordinal scoring: 0, none; 1, <25%; 2, 26%-50%; 3, 51%-75%; 4, >75% of tissue fields. For perivascular infiltrates, the severity-based ordinal scoring was evaluated: 0, absent; 1, minor (solitary to lose infiltration); 2, moderate (small to medium aggregates); 3, severe (robust aggregates that are circumferential around vessels and extend into adjacent parenchyma). For interstitial scores, the severity for the

presence of interstitial disease was assessed: 0, absent; 1, minor (increased cellularity in septa and uncommon inflammatory cells extending into lumen); 3, severe (cellular infiltrates in thickened septa and filling in alveolar with atelectasis and/or diffuse alveolar damage). Final scores were obtained by averaging four fields per mouse.

**Polyfunctional T cell analysis**

Single cell suspension of lungs from IAV-infected mice with DMSO or MSDC treatment were stimulated with PMA (0.1 μg/ml) and Ionomycin (1 μg/ml) (Sigma) for 5 h in the presence of 2μM monensin (BD Biosciences), and then stained with BV711 conjugated anti-CD8 Abs. Cells were fixed and permeabilized by fixation buffer and intracellular staining perm wash buffer (Biolegend, Cat# 420801), and stained with anti-IFN-γ (Biolegend, Cat# 505808), anti-CCL3 (ThermoFisher, Cat# 50-7532-82) and anti-TNF-α (Biolegend, Cat# 506324) Abs for at least 30 min at RT. Samples were processed with flow cytometer.

**Nanostring analysis**

Total RNA from lungs of IAV-infected mice or *in vitro* cultured human AMs with SARS-CoV-2 infection was used for Nanostring analysis. The mRNA expression was analyzed with the nCounter murine immunology panel including 561 targets or the nCounter human myeloid innate immunity panel including 770 targets on the nCounter platform (Nanosting Technologies, Seattle, WA, USA) using 50 ng of total RNA per sample as described previously (*38*). Hybridization reaction was established by following the instruction of manufacture. Aliquots of Reporter Codeset and Capture probeset were thawed at room temperature. Then a master mix was created by adding 70 µl of hybridization buffer to the tube containing the reporter codeset. 8 µl of this master mix was added to each of tubes for different samples, 5 µl (50 ng) of total RNA sample was added into each tube. Then 2 µl of well mixed Capture probeset was added to each tube and placed in the preheated 65°C thermal cycler. All the sample mixes were incubated for 16 hours at 65°C for completion of hybridization. The cartridge with samples were loaded into the NanoString nCounter SPRINT Profiler machine. Data were normalized and analyzed using Nanostring software nSolver 4.0 (Nanosting Technologies). mRNA counts were processed to account for hybridization efficiency, background noise, and sample content, and were normalized using the geometric mean of housekeeping genes. Fold changes were calculated comparing the experimental group to their appropriate controls. Transcript counts less than the

mean of the negative control transcripts for each sample were considered as background. Clustering of differentially expressed genes for the heatmap was done using R language (https://www.r-project.org/). The Nanostring experiments were conducted once using multiple biological samples per group (as indicated in figures).

# Figure S1

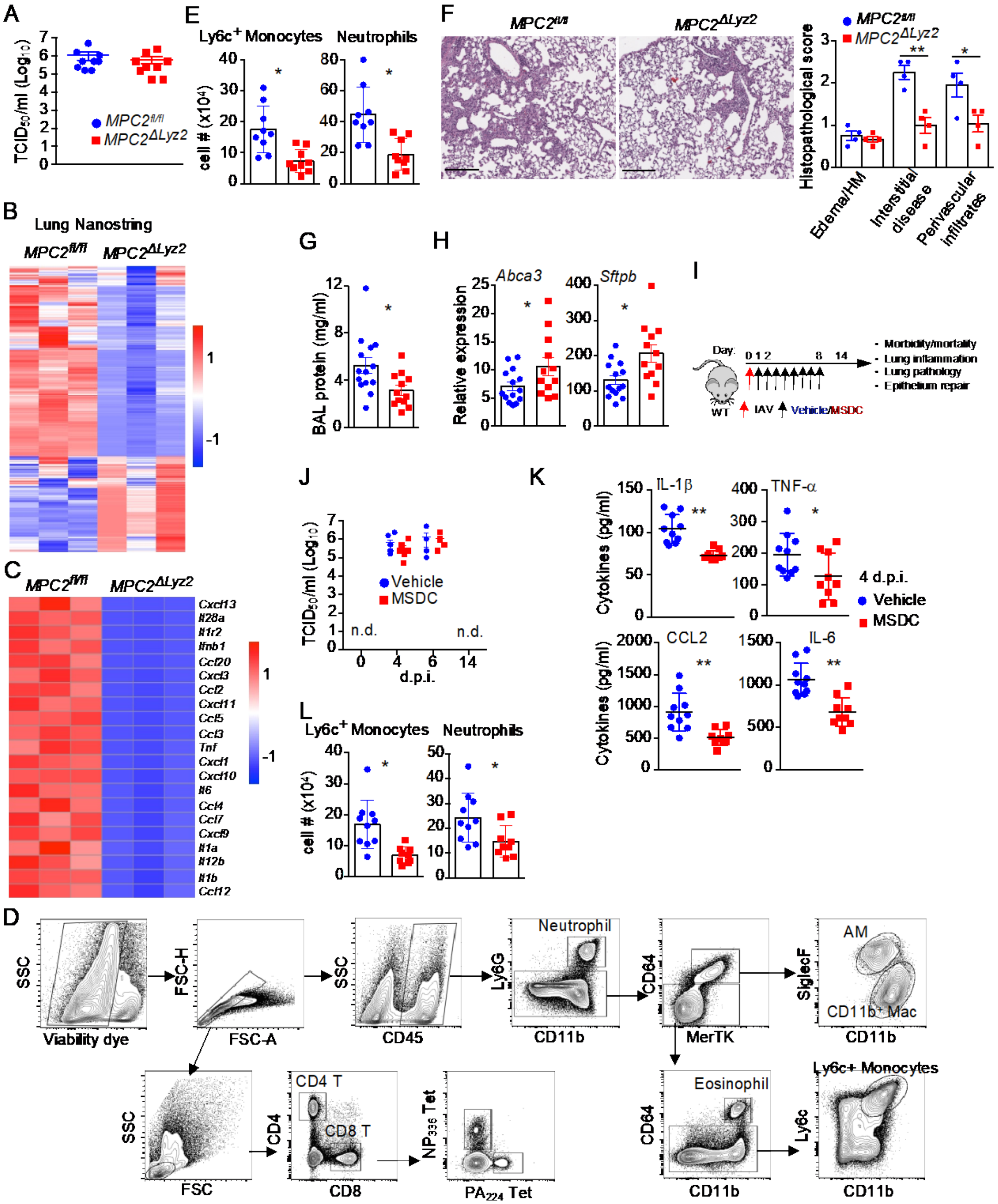

A
TCID50/ml (Log10)
MPC2fl/fl
MPC2ΔLyz2
E
Ly6c+ Monocytes
Neutrophils
cell # (x10⁴)
F
MPC2fl/fl
MPC2ΔLyz2
Histopathological score
Edema/HM
Interstitial disease
Perivascular infiltrates
B
Lung Nanostring
MPC2fl/fl
MPC2ΔLyz2
G
BAL protein (mg/ml)
H
Abca3
Sftpb
Relative expression
I
Day:
WT
IAV
Vehicle/MSDC
Morbidity/mortality
Lung inflammation
Lung pathology
Epithelium repair
C
MPC2fl/fl
MPC2ΔLyz2
Cxcl13
Il28a
Il1r2
Ifnb1
Ccl20
Cxcl3
Ccl2
Cxcl11
Ccl5
Ccl3
Tnf
Cxcl1
Cxcl10
Il6
Ccl4
Ccl7
Cxcl9
Il1a
Il12b
Il1b
Ccl12
J
TCID50/ml (Log10)
n.d.
Vehicle
MSDC
d.p.i.
K
IL-1β
TNF-α
Cytokines (pg/ml)
4 d.p.i.
Vehicle
MSDC
CCL2
IL-6
L
Ly6c+ Monocytes
Neutrophils
cell # (x10⁴)
D
SSC
Viability dye
FSC-H
FSC-A
CD45
Ly6G
CD11b
Neutrophil
CD64
MerTK
SiglecF
AM
CD11b+ Mac
FSC
CD4
CD4 T
CD8 T
CD8
NP336 Tet
PA224 Tet
Eosinophil
Ly6c
Ly6c+ Monocytes

**Fig. S1. Myeloid-specific MPC2-deficiency diminishes IAV-induced lung inflammation.** (**A** to **H**) *MPC2*$^{fl/fl}$ and *MPC2*$^{\Delta Lyz2}$ mice were infected with IAV. (A) BAL viral titers were measured at 4 d.p.i. (B and C) Nanostring analysis of the expression of 560 immune-related genes (Mouse Immunology Panel) in lungs at 4 d.p.i. (n = 3). Heatmap for total differentially expressed genes (B) or differentially expressed cytokine/chemokine genes (C) were shown. (D) Flow cytometry gating strategy for the identification of lung myeloid cells and lymphocytes. (E) Numbers of Ly6c$^{+}$ inflammatory monocytes and neutrophils in the BAL at 4 d.p.i. (F) Representative image of H&E staining of lung section (n = 4). Scale bar, 200 µm (left). Right, the quantification of pathological lesions at 14 d.p.i. HM, hyaline membranes. (G) Protein concentrations in the BAL fluid at 14 d.p.i. (H) *Abca3* and *Sftpb* gene expression in the lungs at 14 d.p.i. (**I** to **L**) C57BL/6 WT mice were infected with IAV and treated with vehicle or MSDC. (I) Schematic diagram. (J) BAL viral titers were measured at the indicated time points (n = 5-10). n.d., not detected. (K) Cytokine levels in the BAL at 4 d.p.i. (n = 9-10). (L) Numbers of Ly6c$^{+}$ inflammatory monocytes and neutrophils in the BAL at 4 d.p.i. (n = 9-10). Pooled data (A, E, G, H, J, K, and L) from at least two independent experiments. Data are presented as means ± SEM. *, $p < 0.05$; **, $p < 0.01$. The *p* value was determined by a two-tailed Student's t-test for (A, E, F, G, H and J to L).

Figure S2

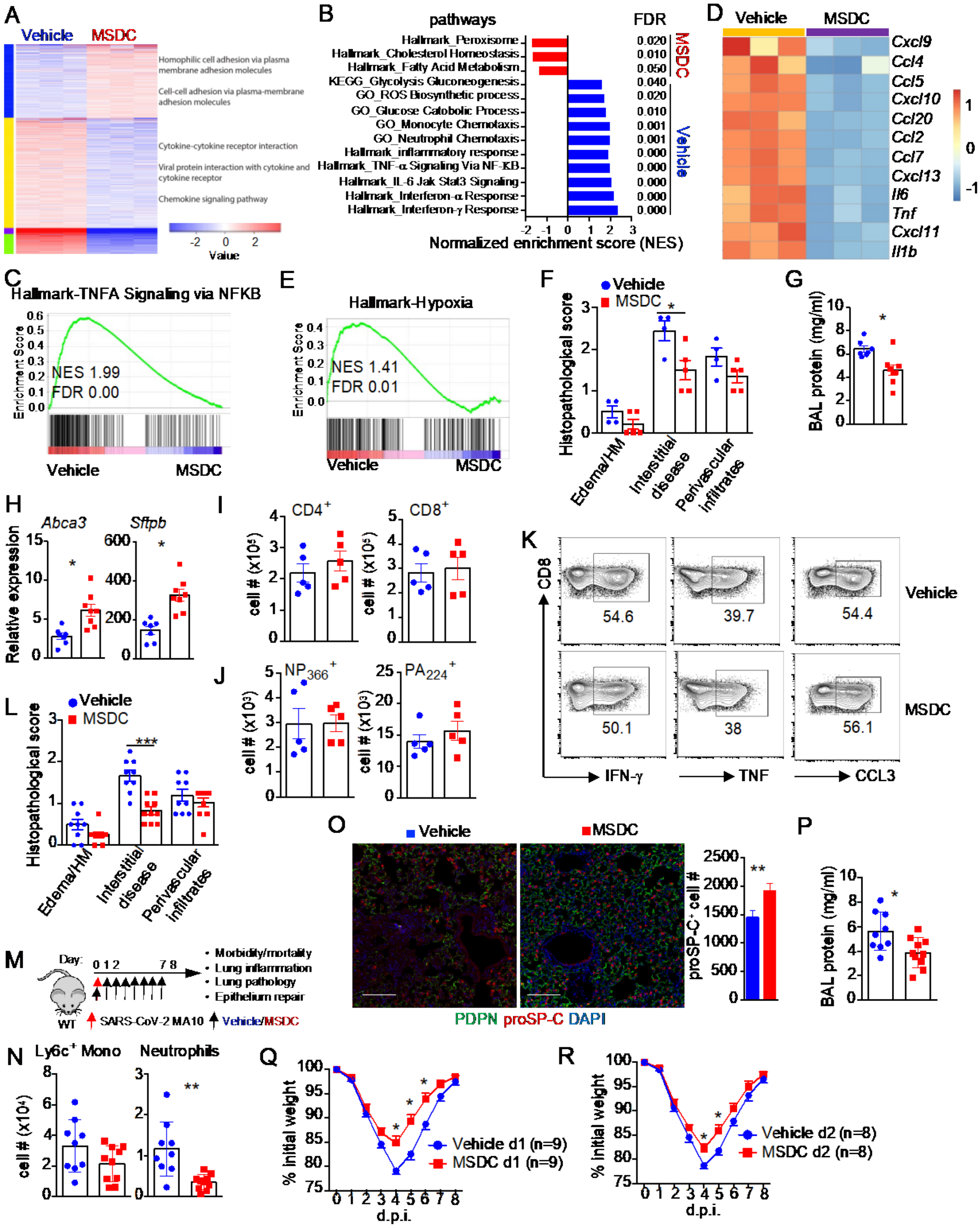
A
Vehicle
MSDC
Homophilic cell adhesion via plasma membrane adhesion molecules
Cell-cell adhesion via plasma-membrane adhesion molecules
Cytokine-cytokine receptor interaction
Viral protein interaction with cytokine and cytokine receptor
Chemokine signaling pathway
Value
B
pathways
FDR
Hallmark_Peroxisome 0.020
Hallmark_Cholesterol Homeostasis 0.010
Hallmark_Fatty Acid Metabolism 0.050
KEGG_Glycolysis Gluconeogenesis 0.040
GO_ROS Biosynthetic process 0.020
GO_Glucose Catabolic Process 0.010
GO_Monocyte Chemotaxis 0.001
GO_Neutrophil Chemotaxis 0.001
Hallmark_inflammatory response 0.000
Hallmark_TNF-α Signaling Via NF-KB 0.000
Hallmark_IL-6 Jak Stat3 Signaling 0.000
Hallmark_Interferon-α Response 0.000
Hallmark_Interferon-γ Response 0.000
MSDC
Vehicle
Normalized enrichment score (NES)
D
Vehicle
MSDC
Cxcl9
Ccl4
Ccl5
Cxcl10
Ccl20
Ccl2
Ccl7
Cxcl13
Il6
Tnf
Cxcl11
Il1b
C
Hallmark-TNFA Signaling via NFKB
Enrichment Score
NES 1.99
FDR 0.00
Vehicle
MSDC
E
Hallmark-Hypoxia
Enrichment Score
NES 1.41
FDR 0.01
Vehicle
MSDC
F
Vehicle
MSDC
Histopathological score
Edema/HM
Interstitial disease
Perivascular infiltrates
G
BAL protein (mg/ml)
H
Abca3
Sftpb
Relative expression
I
CD4+
CD8+
cell # (x10^5)
J
NP366+
PA224+
cell # (x10^3)
K
CD8
54.6
39.7
54.4
Vehicle
50.1
38
56.1
MSDC
IFN-γ
TNF
CCL3
L
Vehicle
MSDC
Histopathological score
Edema/HM
Interstitial disease
Perivascular infiltrates
M
Day:
0 1 2
7 8
WT
• Morbidity/mortality
• Lung inflammation
• Lung pathology
• Epithelium repair
SARS-CoV-2 MA10
Vehicle/MSDC
O
Vehicle
MSDC
PDPN proSP-C DAPI
proSP-C+ cell #
P
BAL protein (mg/ml)
N
Ly6c+ Mono
Neutrophils
cell # (x10^4)
Q
% initial weight
Vehicle d1 (n=9)
MSDC d1 (n=9)
d.p.i.
R
% initial weight
Vehicle d2 (n=8)
MSDC d2 (n=8)
d.p.i.

**Fig. S2. MSDC treatment decreases pulmonary inflammation without altering host antiviral immunity following IAV or SARS-CoV-2 infection.**

(**A** to **K**) C57BL/6 WT mice were infected with IAV and treated with vehicle or MSDC. (**A** to **E**) RNA-seq analysis of lungs at 4 d.p.i. (n = 3). (A) Heatmap of differentially expressed genes. (B) Normalized enrichment scores of gene sets from Hallmark, GO, and KEGG dataset. (C) GSEA of TNF signaling gene set was shown. (D) Heatmap showing differentially expressed cytokines. (E) GSEA of hypoxia gene set was shown. (**F**) The quantification of pathological lesions at 14 d.p.i. for Fig. 1I. HM, hyaline membranes. (**G**) Protein concentrations in the BAL at 14 d.p.i. (n = 7-8). (**H**) *Abca3* and *Sftpb* gene expression in the lungs at 14 d.p.i. (n = 7-8). (**I** to **K**) The antiviral immunity mediated by adaptive immune response was measured at 6 d.p.i. (n = 5). Lung total $CD4^+$ and $CD8^+$ T cells (I), and NP336 and PA224 $tetramer^+CD8^+$ T cells (J) were enumerated. (K) Representative flow cytometry plots of IFN-γ, TNF and CCL3 production in polyclonal $CD8^+$ T cells after PMA and ionomycin stimulation at 6 d.p.i. (**L** to **P**) C57BL/6 WT mice were infected with SARS-CoV-2 MA10 virus and treated with vehicle or MSDC (n = 9-10). (L) The quantification of pathological lesions at 8 d.p.i. for Fig. 1L. HM, hyaline membranes. (M) Schematic diagram. (N) Numbers of $Ly6c^+$ inflammatory monocytes and neutrophils in the BAL at 8 d.p.i. (O) Fluorescence microscopy images of PDPN (marker for alveolar type I epithelial cells), proSP-C (marker for alveolar type II epithelial cells) and DAPI staining in fixed lung tissues at 8 d.p.i (n=5). Scale bar, 50 µm. Quantification of $proSP\text{-}C^+$ cell number was performed using at least 10 random fields (10x) of alveolar space per mouse lung. (P) Protein concentrations in the BAL at 8 d.p.i. (**Q** and **R**) WT mice were infected with SARS-CoV-2 MA10 virus and treated with vehicle or MSDC from day 1 (Q) or day 2 (R) post infection. Pooled data (G, H, N, and P to R) from at least two independent experiments. Data are presented as means ± SEM. *, $p < 0.05$; **, $p < 0.01$. The $p$ value was determined by a two-tailed Student's t-test (F, G, H, I, J, L, N, and P) or multiple t-tests (Q and R).

## Figure S3

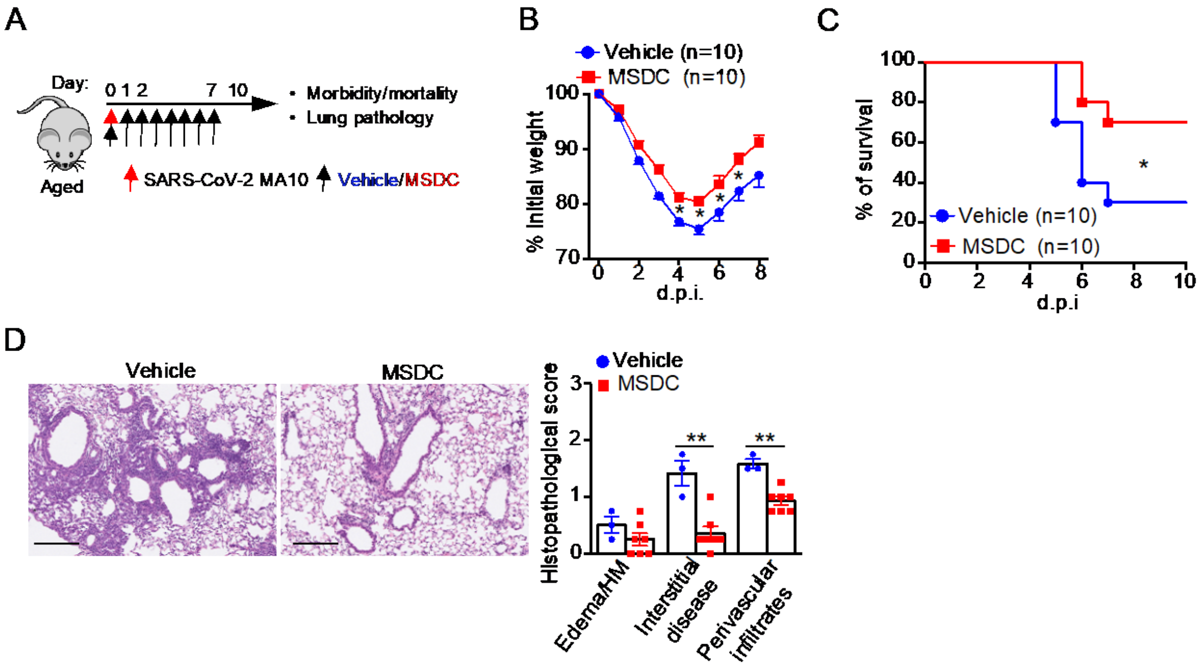


**Fig. S3. MSDC treatment decreases host morbidity and mortality following SARS-CoV-2 infection in aged mice.**

(**A** to **D**) Aged (13-14-month-old) C57BL/6 mice were infected with $8x10^4$ PFU of SARS-CoV-2 MA10 and treated with vehicle or MSDC. (A) Schematic diagram. Host morbidity (B) and mortality (C) were monitored. (D) H&E staining of lung section (n = 3-7) at 10 d.p.i. Scale bar, 200 µm. Right, the quantification of pathological lesions. HM, hyaline membranes. Pooled data (B and C) from two independent experiments. Data are presented as means ± SEM. *, $p < 0.05$; **, $p < 0.01$. The $p$ value was determined by multiple t-tests (B), Logrank test (C) or a two-tailed Student's t-test (D).

# Figure S4

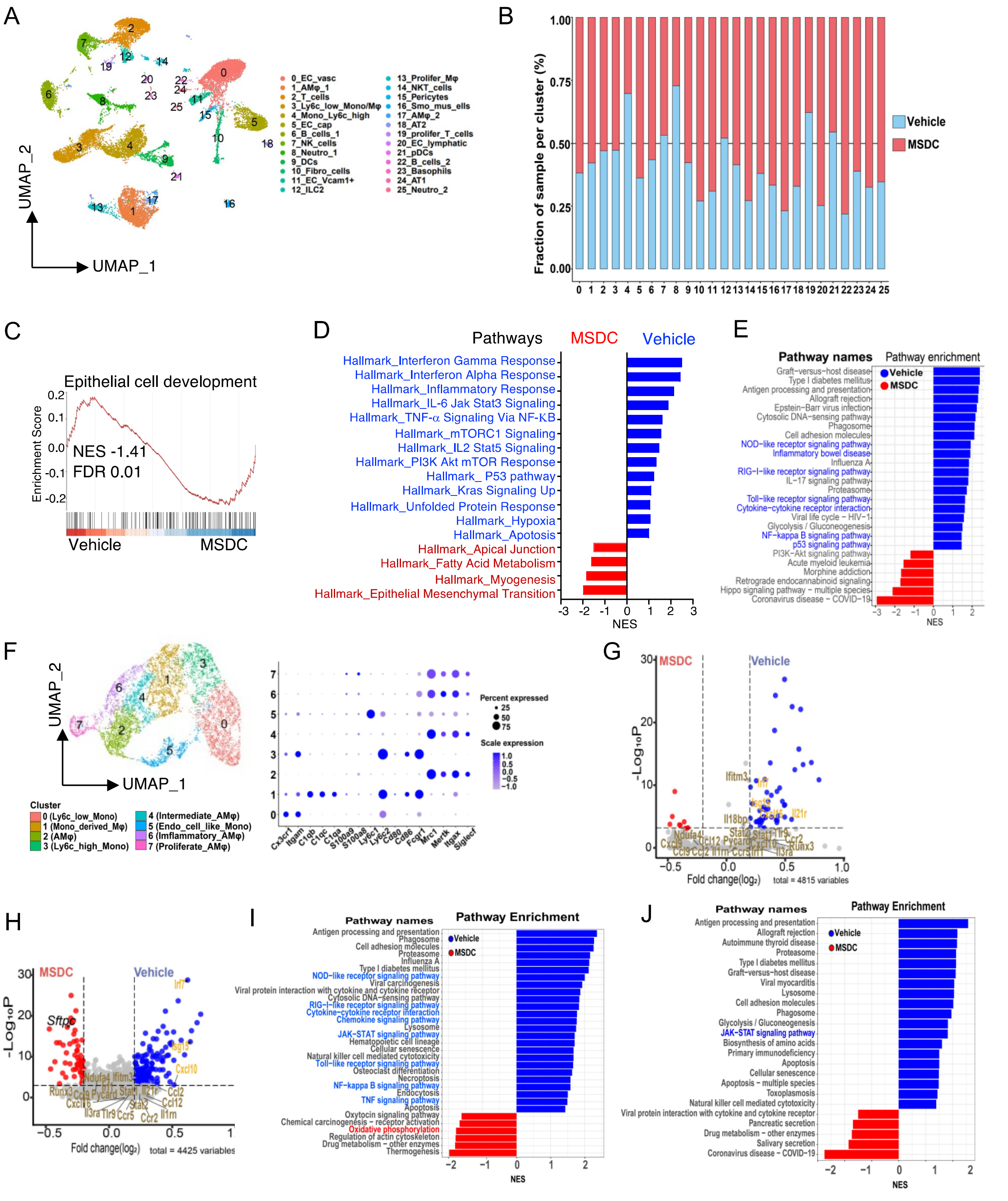
A
UMAP_1
UMAP_2
0_EC_vasc
1_AMφ_1
2_T_cells
3_Ly6c_low_Mono/Mφ
4_Mono_Ly6c_high
5_EC_cap
6_B_cells_1
7_NK_cells
8_Neutro_1
9_DCs
10_Fibro_cells
11_EC_Vcam1+
12_ILC2
13_Prolifer_Mφ
14_NKT_cells
15_Pericytes
16_Smo_mus_ells
17_AMφ_2
18_AT2
19_prolifer_T_cells
20_EC_lymphatic
21_pDCs
22_B_cells_2
23_Basophils
24_AT1
25_Neutro_2
B
Fraction of sample per cluster (%)
Vehicle
MSDC
C
Epithelial cell development
Enrichment Score
NES -1.41
FDR 0.01
Vehicle
MSDC
D
Pathways
MSDC
Vehicle
Hallmark_Interferon Gamma Response
Hallmark_Interferon Alpha Response
Hallmark_Inflammatory Response
Hallmark_IL-6 Jak Stat3 Signaling
Hallmark_TNF-α Signaling Via NF-KB
Hallmark_mTORC1 Signaling
Hallmark_IL2 Stat5 Signaling
Hallmark_PI3K Akt mTOR Response
Hallmark_ P53 pathway
Hallmark_Kras Signaling Up
Hallmark_Unfolded Protein Response
Hallmark_Hypoxia
Hallmark_Apotosis
Hallmark_Apical Junction
Hallmark_Fatty Acid Metabolism
Hallmark_Myogenesis
Hallmark_Epithelial Mesenchymal Transition
NES
E
Pathway names
Pathway enrichment
Graft-versus-host disease
Type I diabetes mellitus
Antigen processing and presentation
Allograft rejection
Epstein-Barr virus infection
Cytosolic DNA-sensing pathway
Phagosome
Cell adhesion molecules
NOD-like receptor signaling pathway
Inflammatory bowel disease
Influenza A
RIG-I-like receptor signaling pathway
IL-17 signaling pathway
Proteasome
Toll-like receptor signaling pathway
Cytokine-cytokine receptor interaction
Viral life cycle - HIV-1
Glycolysis / Gluconeogenesis
NF-kappa B signaling pathway
p53 signaling pathway
PI3K-Akt signaling pathway
Acute myeloid leukemia
Morphine addiction
Retrograde endocannabinoid signaling
Hippo signaling pathway - multiple species
Coronavirus disease - COVID-19
NES
F
UMAP_1
UMAP_2
Cluster
0 (Ly6c_low_Mono)
1 (Mono_derived_Mφ)
2 (AMφ)
3 (Ly6c_high_Mono)
4 (Intermediate_AMφ)
5 (Endo_cell_like_Mono)
6 (Imflammatory_AMφ)
7 (Proliferate_AMφ)
Percent expressed
Scale expression
Cx3cr1
Itgam
C1qb
C1qc
C1qa
S100a9
S100a8
Ly6c1
Ly6c2
Cd80
Cd86
Fcgr1
Mrc1
Mertk
Itgax
Siglecf
G
MSDC
Vehicle
-Log₁₀P
Fold change(log₂)
total = 4815 variables
H
MSDC
Vehicle
Sftpc
Irf7
Isg15
Cxcl10
Fold change(log₂)
total = 4425 variables
I
Pathway names
Pathway Enrichment
Antigen processing and presentation
Phagosome
Cell adhesion molecules
Proteasome
Influenza A
Type I diabetes mellitus
NOD-like receptor signaling pathway
Viral carcinogenesis
Viral protein interaction with cytokine and cytokine receptor
Cytosolic DNA-sensing pathway
RIG-I-like receptor signaling pathway
Cytokine-cytokine receptor interaction
Chemokine signaling pathway
Lysosome
JAK-STAT signaling pathway
Hematopoietic cell lineage
Cellular senescence
Natural killer cell mediated cytotoxicity
Toll-like receptor signaling pathway
Osteoclast differentiation
Necroptosis
NF-kappa B signaling pathway
Endocytosis
TNF signaling pathway
Apoptosis
Oxytocin signaling pathway
Chemical carcinogenesis - receptor activation
Oxidative phosphorylation
Regulation of actin cytoskeleton
Drug metabolism - other enzymes
Thermogenesis
NES
J
Pathway names
Pathway Enrichment
Antigen processing and presentation
Allograft rejection
Autoimmune thyroid disease
Proteasome
Type I diabetes mellitus
Graft-versus-host disease
Viral myocarditis
Lysosome
Cell adhesion molecules
Phagosome
Glycolysis / Gluconeogenesis
JAK-STAT signaling pathway
Biosynthesis of amino acids
Primary immunodeficiency
Apoptosis
Cellular senescence
Apoptosis - multiple species
Toxoplasmosis
Natural killer cell mediated cytotoxicity
Viral protein interaction with cytokine and cytokine receptor
Pancreatic secretion
Drug metabolism - other enzymes
Salivary secretion
Coronavirus disease - COVID-19
NES

**Fig. S4. scRNA-seq analysis of IAV-infected lungs with or without MSDC treatment.**

(**A** to **J**) C57BL/6 WT mice were infected with IAV and treated with vehicle or MSDC. Lung cells were pooled from three individual mice from each group at 4 d.p.i, and subjected to scRNA-seq analysis. (A) UMAP plot visualization of major cell types across vehicle- or MSDC-treated lung cells. (B) The bar plot showing the relative contributions of each cluster by each studied subject. (C) GSEA of Epithelial cell development gene set was shown for lung epithelial cells (cluster 18 and 24). A negative normalized enrichment score (NES) value indicates enrichment in MSDC treatment lung cells. (D) Normalized enrichment scores of gene sets in lung cells from Hallmark dataset. (E) Normalized enrichment scores of gene sets in lung epithelial cells (cluster 18 and 24) from KEGG pathways. (F) UMAP showing clusters of monocytes and macrophages from (A) in vehicle- or MSDC- treated lung cells (left panel). A dot plot showing the percentage of cells expressing the selected marker genes using dot size and the average expression level of the genes in different clusters (right panel). (G and H) Volcano plot showing the differentially expressed genes in monocytes (cluster 0 and 3) (G) or monocyte derived macrophages (cluster 1) (H) of vehicle (blue) and MSDC (red) treated group. (I and J) Normalized enrichment scores of gene sets in lung AMs (cluster 2, 4, 6 and 7) (I) and combined monocytes and monocyte derived macrophages (cluster 0, 1 and 3) (J) from KEGG pathways.

# Figure S5

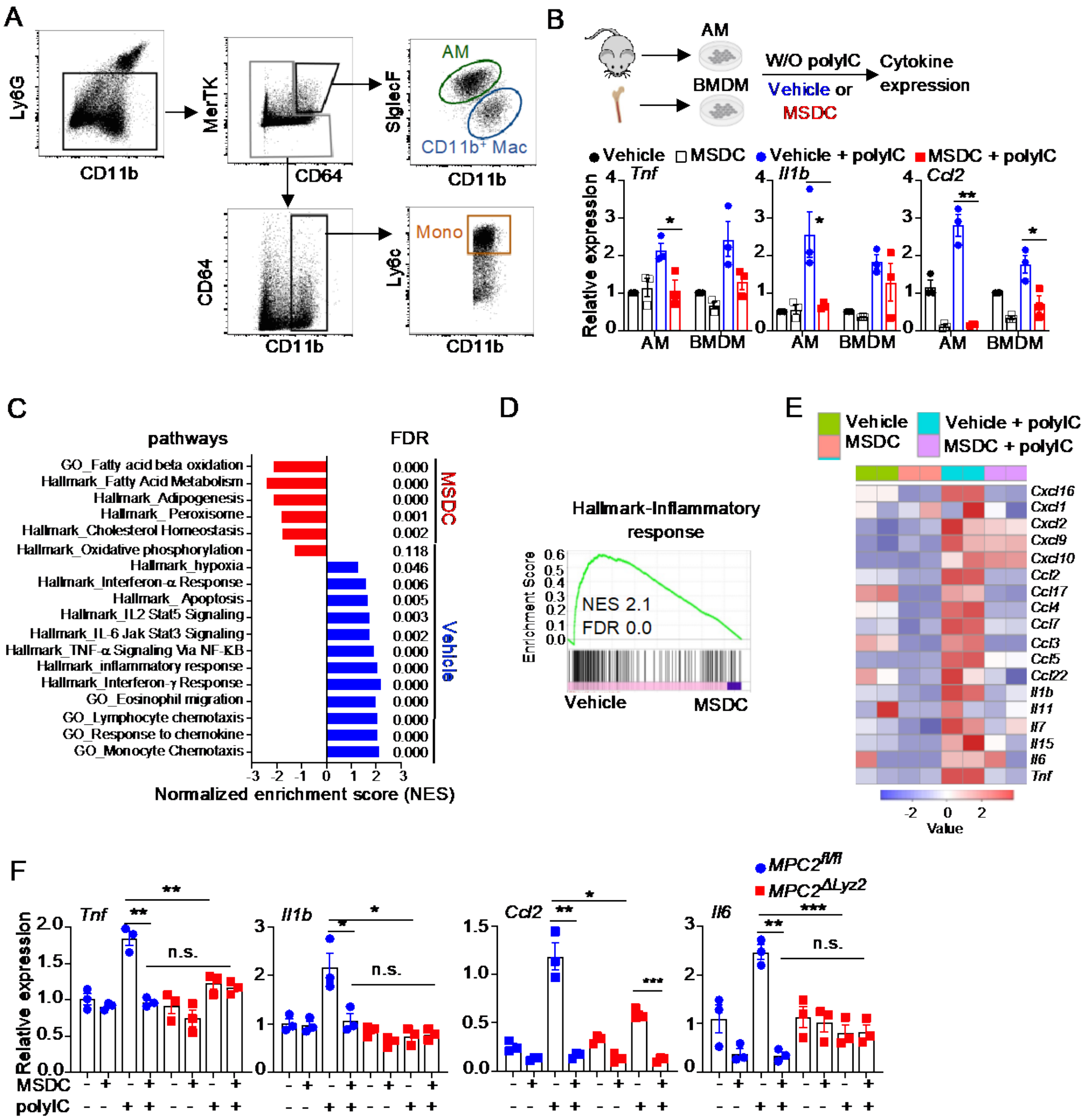

A
Ly6G
CD11b
MerTK
CD64
SiglecF
AM
CD11b+ Mac
Mono
Ly6c
B
AM
BMDM
W/O polyIC
Vehicle or
MSDC
Cytokine
expression
Vehicle
MSDC
Vehicle + polyIC
MSDC + polyIC
Relative expression
Tnf
Il1b
Ccl2
C
pathways
FDR
GO_Fatty acid beta oxidation 0.000
Hallmark_Fatty Acid Metabolism 0.000
Hallmark_Adipogenesis 0.000
Hallmark_ Peroxisome 0.001
Hallmark_Cholesterol Homeostasis 0.002
Hallmark_Oxidative phosphorylation 0.118
Hallmark_hypoxia 0.046
Hallmark_Interferon-α Response 0.006
Hallmark_ Apoptosis 0.005
Hallmark_IL2 Stat5 Signaling 0.003
Hallmark_IL-6 Jak Stat3 Signaling 0.002
Hallmark_TNF-α Signaling Via NF-KB 0.000
Hallmark_inflammatory response 0.000
Hallmark_Interferon-γ Response 0.000
GO_Eosinophil migration 0.000
GO_Lymphocyte chemotaxis 0.000
GO_Response to chemokine 0.000
GO_Monocyte Chemotaxis 0.000
MSDC
Vehicle
Normalized enrichment score (NES)
D
Hallmark-Inflammatory response
Enrichment Score
NES 2.1
FDR 0.0
Vehicle
MSDC
E
Vehicle
MSDC
Vehicle + polyIC
MSDC + polyIC
Cxcl16
Cxcl1
Cxcl2
Cxcl9
Cxcl10
Ccl2
Ccl17
Ccl4
Ccl7
Ccl3
Ccl5
Ccl22
Il1b
Il11
Il7
Il15
Il6
Tnf
Value
F
MPC2fl/fl
MPC2ΔLyz2
Relative expression
Tnf
Il1b
Ccl2
Il6
n.s.
MSDC
polyIC

**Fig. S5. MSDC treatment reduces lung macrophage inflammation following IAV infection.** (**A**) Flow cytometry gating strategy for sorting alveolar macrophages (AM), $CD11b^{+}$ macrophages ($CD11b^{+}$ Mac) and monocytes (Mono). (**B**) Relative inflammatory gene expression in mouse AMs or BMDM stimulated with or without Poly IC in the presence of vehicle or MSDC overnight *in vitro*. (**C** to **E**) RNA-seq analysis of mouse AMs stimulated with or without Poly IC in the presence of vehicle or MSDC overnight *in vitro*. (C) Normalized enrichment scores of gene sets in Poly IC stimulated AMs with vehicle or MSDC treatment from Hallmark and GO dataset. (D) GSEA of Inflammatory response gene set was shown for Poly IC stimulated AMs with vehicle or MSDC treatment. (E) Heatmap for differentially expressed cytokine/chemokine genes were shown. (**F**) *Tnf*, *Il1b*, *Ccl2*, and *Il6* gene expression in WT ($MPC2^{fl/fl}$) or MPC2-deficient ($MPC2^{\Delta Lyz2}$) AMs with indicted treatment overnight *in vitro*. Data are presented as means ± SEM. *, $p < 0.05$; **, $p < 0.01$; ***, $p < 0.001$. The $p$ value was determined by one-way ANOVA (B and F).

Figure S6

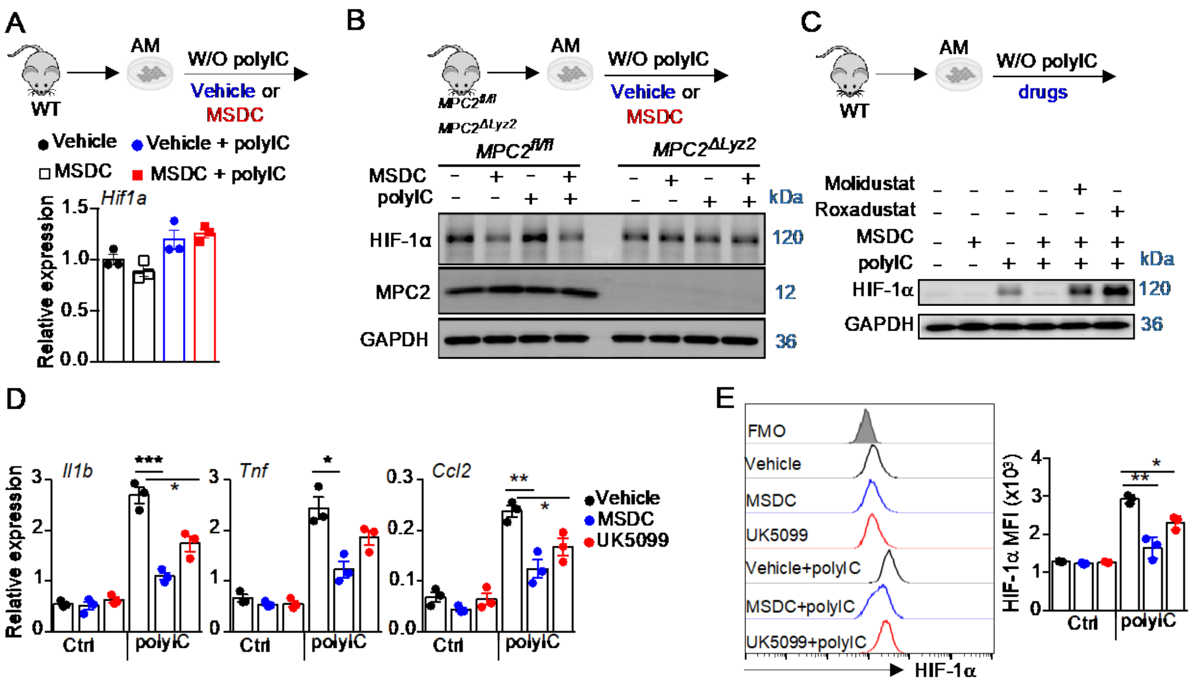


**Fig. S6. MPC inhibition decreases HIF-1α levels in lung macrophages.**

(**A**) The mRNA levels of *Hif1a* in AMs stimulated with or without Poly IC in the presence of vehicle or MSDC overnight *in vitro*. (**B**) HIF-1α and MPC2 protein levels in WT ($MPC2^{fl/fl}$) or MPC2-deficient ($MPC2^{\Delta Lyz2}$) AMs stimulated with or without Poly IC in the presence of vehicle or MSDC overnight *in vitro*. (**C**) HIF-1α protein levels in AMs with indicted treatment overnight *in vitro*. (**D** and **E**) *Il1b*, *Tnf* and *Ccl2* gene expression (D) and HIF-1α levels (E) in AMs stimulated with or without Poly IC in the presence of MSDC, UK5099 or vehicle overnight *in vitro*. Data are presented as means ± SEM. *, $p < 0.05$; **, $p < 0.01$; ***, $p < 0.001$. The $p$ value was determined by one-way ANOVA (D and E).

Figure S7

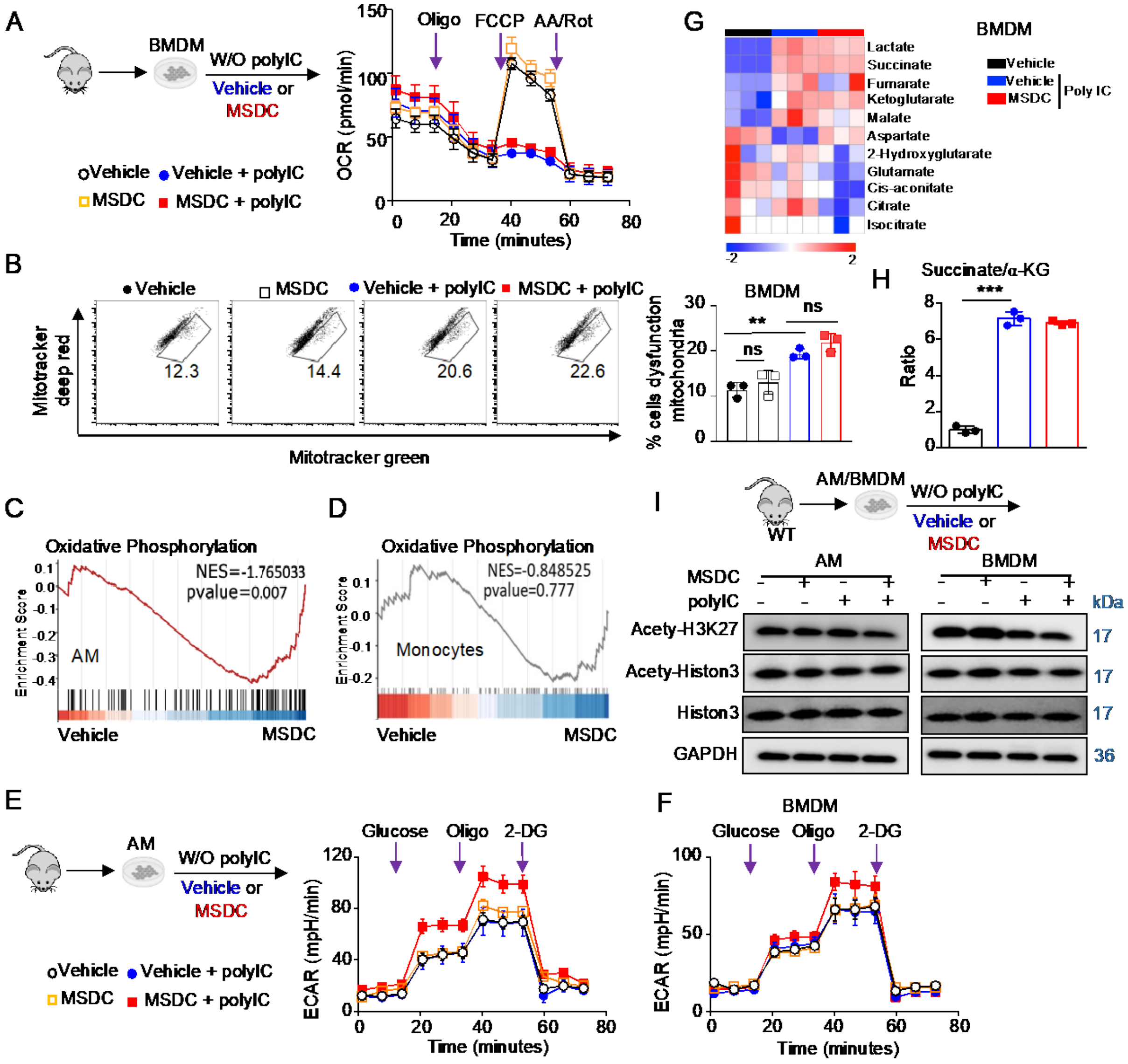

A
BMDM
W/O polyIC
Vehicle or
MSDC
Vehicle
Vehicle + polyIC
MSDC
MSDC + polyIC
Oligo
FCCP
AA/Rot
OCR (pmol/min)
Time (minutes)
B
Vehicle
MSDC
Vehicle + polyIC
MSDC + polyIC
Mitotracker deep red
Mitotracker green
12.3
14.4
20.6
22.6
BMDM
% cells dysfunction mitochondria
C
Oxidative Phosphorylation
NES=-1.765033
pvalue=0.007
AM
Enrichment Score
Vehicle
MSDC
D
Oxidative Phosphorylation
NES=-0.848525
pvalue=0.777
Monocytes
Enrichment Score
Vehicle
MSDC
E
AM
W/O polyIC
Vehicle or
MSDC
Vehicle
Vehicle + polyIC
MSDC
MSDC + polyIC
Glucose
Oligo
2-DG
ECAR (mpH/min)
Time (minutes)
F
BMDM
Glucose
Oligo
2-DG
ECAR (mpH/min)
Time (minutes)
G
BMDM
Lactate
Succinate
Fumarate
Ketoglutarate
Malate
Aspartate
2-Hydroxyglutarate
Glutamate
Cis-aconitate
Citrate
Isocitrate
Vehicle
Vehicle
MSDC
Poly IC
H
Succinate/α-KG
Ratio
I
AM/BMDM
WT
W/O polyIC
Vehicle or
MSDC
AM
BMDM
MSDC
polyIC
kDa
Acety-H3K27
Acety-Histon3
Histon3
GAPDH
17
17
17
36

**Fig. S7. MPC inhibition does not alter mitochondrial fitness in BMDM.**

(**A** and **B**) BMDM were stimulated with or without Poly IC in the presence of vehicle or MSDC overnight *in vitro*. (A) OCR was measured in BMDM. (B) Flow cytometry showing mitochondrial mass by Mitotracker green versus Mitotracker deep red in BMDM and quantification on the right. (**C** and **D**) GSEA of Oxidative Phosphorylation gene set was shown for Poly IC stimulated AMs (C) and BMDM (D) with vehicle or MSDC treatment. (**E** and **F**) ECAR was measured in AMs (E) and BMDM (F) stimulated with or without Poly IC in the presence of vehicle or MSDC overnight *in vitro*. (**G**) Heatmap showing TCA cycle metabolites measured in BMDMs (n = 3). (**H**) Succinate to Ketoglutarate (α-KG) ratios in (G). (**I**) Immunoblot analysis of K27-acetylated histone3 (Acety-H3K27), acetylated histon3 (Acety-Histon3) and histon3 protein levels in AMs (left) and BMDM (right) stimulated with or without Poly IC in the presence of vehicle or MSDC overnight *in vitro*. Data are presented as means ± SEM. **, $p < 0.01$. The $p$ value was determined by one-way ANOVA (B) and a two-tailed Student's t-test (H).

Figure S8

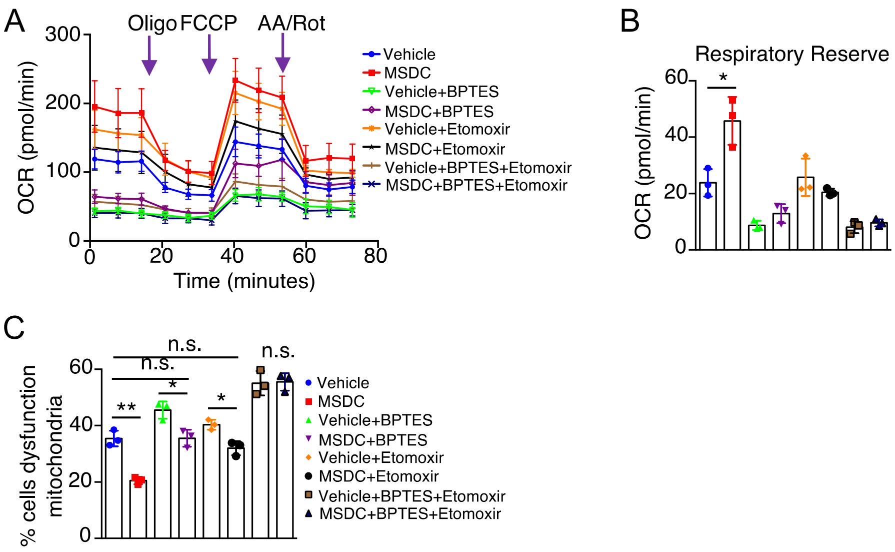


**Fig. S8. Utilization of fatty acid or glutamine oxidation in AMs with MPC inhibition.** (**A** to **C**) AMs were stimulated with Poly IC in the presence of vehicle or MSDC combined with BPTES and/or Etomoxir overnight *in vitro*. OCR of AMs (A) and quantification of respiratory reverse (B). Quantification of flow cytometry showing mitochondrial mass by Mitotracker green versus Mitotracker deep red in AMs (C). Data are presented as means ± SEM. *, $p < 0.05$; **, $p < 0.01$. The $p$ value was determined by one-way ANOVA (B and C).

# Figure S9

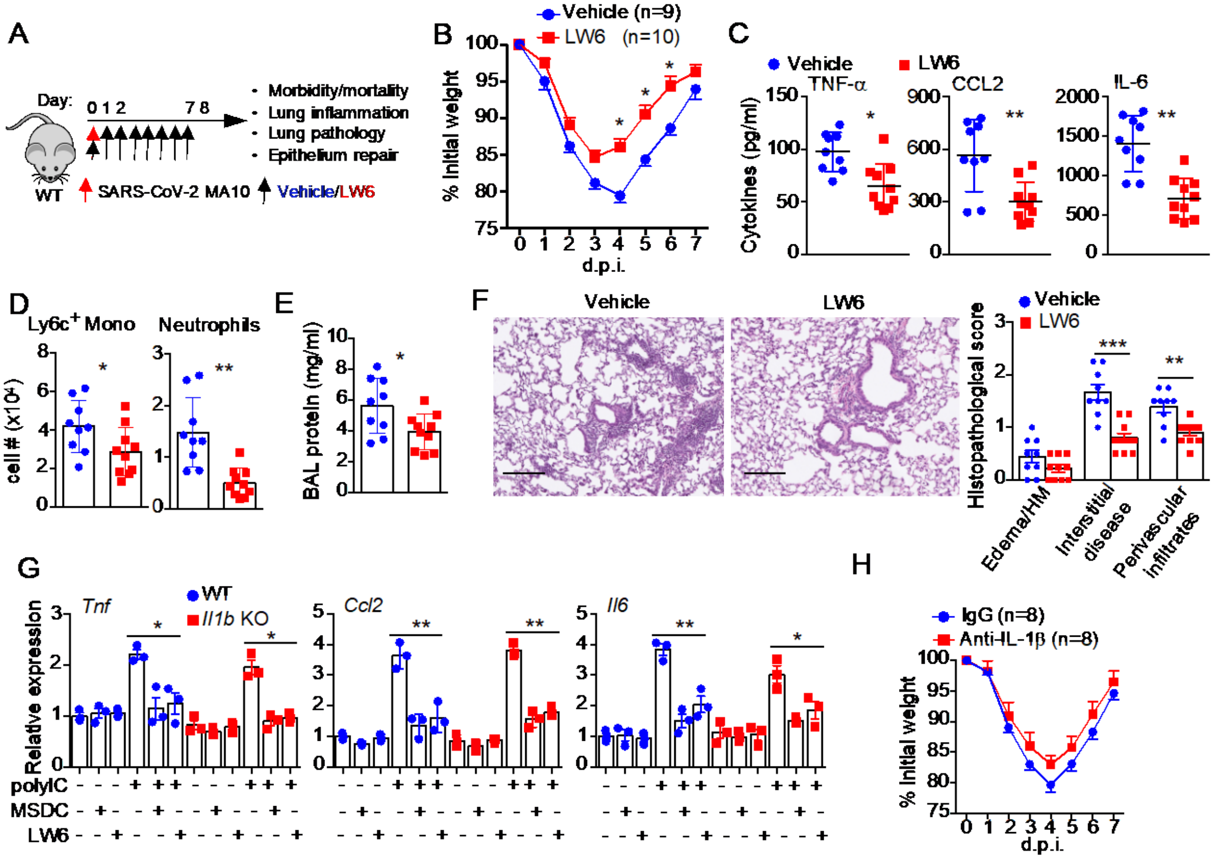

A
Day: 0 1 2 7 8
Morbidity/mortality
Lung inflammation
Lung pathology
Epithelium repair
WT
SARS-CoV-2 MA10
Vehicle/LW6
B
Vehicle (n=9)
LW6 (n=10)
% Initial weight
d.p.i.
C
Vehicle
LW6
TNF-α
CCL2
IL-6
Cytokines (pg/ml)
D
Ly6c+ Mono
Neutrophils
cell # (x10⁴)
E
BAL protein (mg/ml)
F
Vehicle
LW6
Histopathological score
Edema/HM
Interstitial disease
Perivascular infiltrates
G
Tnf
Ccl2
Il6
WT
Il1b KO
Relative expression
polyIC
MSDC
LW6
H
IgG (n=8)
Anti-IL-1β (n=8)
% Initial weight
d.p.i.

**Fig. S9. HIF-1α inhibition ameliorates SARS-CoV-2-caused pulmonary inflammation.** (**A** to **F**) C57BL/6 WT mice were infected with SARS-CoV-2 MA10 virus and treated with vehicle or HIF-1α inhibitor LW6 (n = 9-10). (A) Schematic diagram. (B) Host morbidity was monitored. (C) BAL cytokine levels at 8 d.p.i. (D) Numbers of $Ly6c^+$ inflammatory monocytes and neutrophils in the BAL at 8 d.p.i. (E) Protein concentrations in the BAL at 8 d.p.i. (F) Representative image of H&E staining of lung section at 8 d.p.i. Scale bar, 200 μm (left). Right, the quantification of pathological lesions. HM, hyaline membranes. (**G**) *Tnf* , *Ccl2* and *Il6* gene expression in AMs from WT or *Il1b* ko mice that stimulated with or without Poly IC in the presence of MSDC, LW6 or vehicle overnight *in vitro*. (**H**) C57BL/6 WT mice were infected with SARS-CoV-2 MA10 virus, and were administered by intraperitoneal with 400 μg IgG or Anti-IL-1β antibodies at day 1 and day 3 post infection. Pooled data (B to E, and H) from two independent experiments. Data are presented as means ± SEM. *, $p < 0.05$. **, $p < 0.01$. The $p$ value was determined by multiple t-tests (B and H), a two-tailed Student's t-test (C to F) or one-way ANOVA (G).

Figure S10

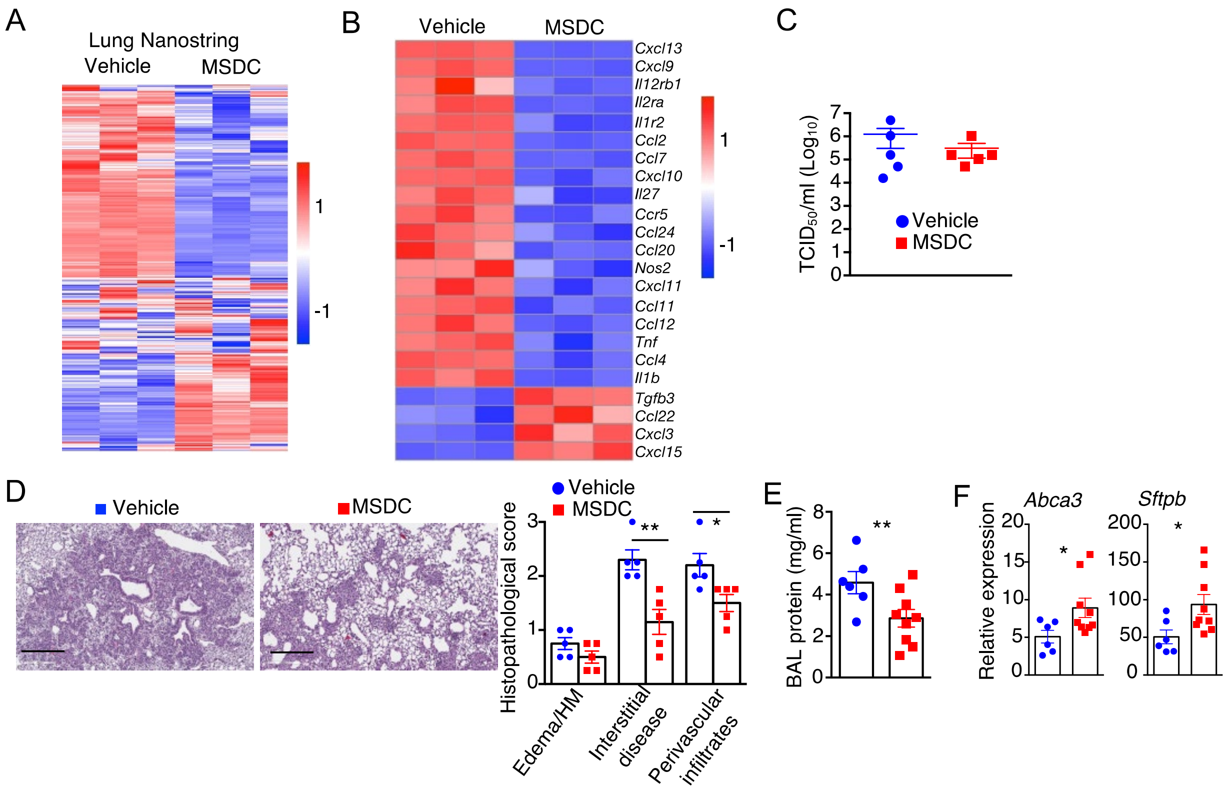


**Fig. S10. MSDC treatment ameliorates lung inflammation and promotes tissue recovery following IAV infection in DIO mice.**

(**A** to **F**) DIO mice were infected with IAV and treated with vehicle or MSDC. (A and B) Nanostring analysis of the expression of 560 immune-related genes (Mouse Immunology Panel) in lungs at 5 d.p.i. (n = 3). Heatmap for total differentially expressed genes (A) or differentially expressed cytokine/chemokine genes (B) were shown. (C) BAL viral titers were measured at 5 d.p.i. (n = 5). (D) Representative image of H&E staining of lung section (n = 5) at 15 d.p.i. Scale bar, 200 µm (left). Right, the quantification of pathological lesions. HM, hyaline membranes. (E) Protein concentrations in the BAL at 15 d.p.i. (n = 6-9). (F) *Abca3* and *Sftpb* gene expression in the lungs at 15 d.p.i. (n = 6-9). Pooled data (E and F) from two independent experiments. Data are presented as means ± SEM. *, $p < 0.05$; **, $p < 0.01$. The $p$ value was determined by a two-tailed Student's t-test (C to F).

# Figure S11

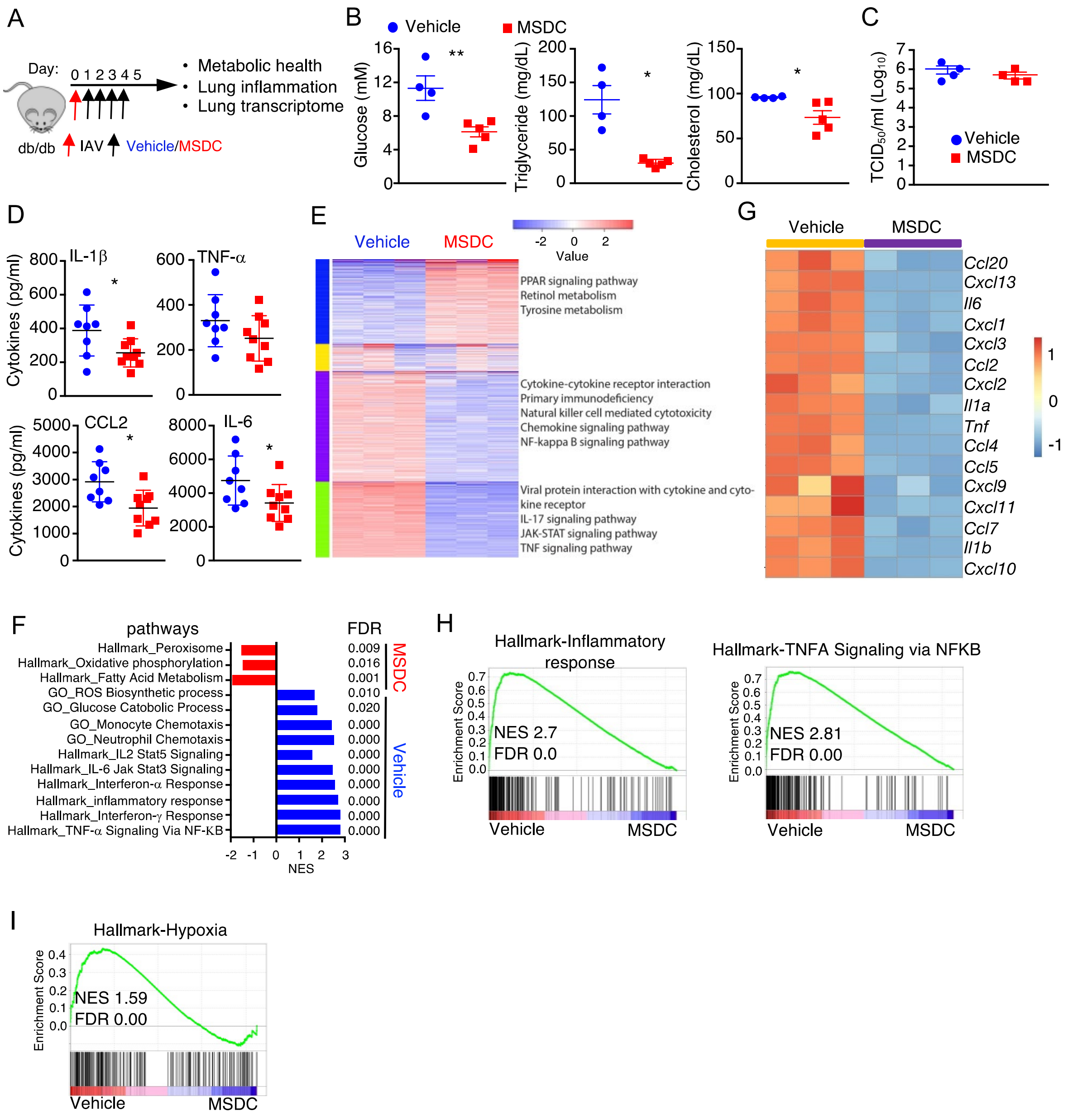
A
Day: 0 1 2 3 4 5
Metabolic health
Lung inflammation
Lung transcriptome
db/db
IAV
Vehicle/MSDC
B
Vehicle
MSDC
Glucose (mM)
Triglyceride (mg/dL)
Cholesterol (mg/dL)
C
TCID50/ml (Log10)
Vehicle
MSDC
D
Cytokines (pg/ml)
IL-1β
TNF-α
CCL2
IL-6
E
Vehicle
MSDC
Value
PPAR signaling pathway
Retinol metabolism
Tyrosine metabolism
Cytokine-cytokine receptor interaction
Primary immunodeficiency
Natural killer cell mediated cytotoxicity
Chemokine signaling pathway
NF-kappa B signaling pathway
Viral protein interaction with cytokine and cyto-kine receptor
IL-17 signaling pathway
JAK-STAT signaling pathway
TNF signaling pathway
G
Vehicle
MSDC
Ccl20
Cxcl13
Il6
Cxcl1
Cxcl3
Ccl2
Cxcl2
Il1a
Tnf
Ccl4
Ccl5
Cxcl9
Cxcl11
Ccl7
Il1b
Cxcl10
F
pathways
FDR
Hallmark_Peroxisome 0.009
Hallmark_Oxidative phosphorylation 0.016
Hallmark_Fatty Acid Metabolism 0.001
GO_ROS Biosynthetic process 0.010
GO_Glucose Catabolic Process 0.020
GO_Monocyte Chemotaxis 0.000
GO_Neutrophil Chemotaxis 0.000
Hallmark_IL2 Stat5 Signaling 0.000
Hallmark_IL-6 Jak Stat3 Signaling 0.000
Hallmark_Interferon-α Response 0.000
Hallmark_inflammatory response 0.000
Hallmark_Interferon-γ Response 0.000
Hallmark_TNF-α Signaling Via NF-KB 0.000
MSDC
Vehicle
NES
H
Hallmark-Inflammatory response
Enrichment Score
NES 2.7
FDR 0.0
Vehicle
MSDC
Hallmark-TNFA Signaling via NFKB
NES 2.81
FDR 0.00
Vehicle
MSDC
I
Hallmark-Hypoxia
Enrichment Score
NES 1.59
FDR 0.00
Vehicle
MSDC

**Fig. S11. MSDC treatment simultaneously targets hyperglycemia and hyper-inflammation following IAV infection in db/db mice.**

(**A** to **I**) The db/db mice were infected with IAV and treated with vehicle or MSDC for 5 days. (A) Schematic diagram. (B) The concentrations of glucose, triglyceride and total cholesterol in the blood (n = 4-5) at 5 d.p.i. (C) BAL viral titers were measured (n = 4) at 5 d.p.i. (D) BAL cytokine levels (n = 8-9) at 5 d.p.i. (E-I) RNA-seq analysis of lungs (n = 3) at 5 d.p.i. (E) Heatmap of differentially expressed genes. (F) Normalized enrichment scores of gene sets from Hallmark and GO dataset. (G) Heatmap showing differentially expressed cytokines. (H and I) GSEA of inflammatory response or TNF signaling (H) and hypoxia (I) gene sets were shown. Representative (B) or pooled data (D) from two independent experiments. Data are presented as means ± SEM *, $p < 0.05$; **, $p < 0.01$. The $p$ value was determined by a two-tailed Student's t-test (B to D).

Figure S12

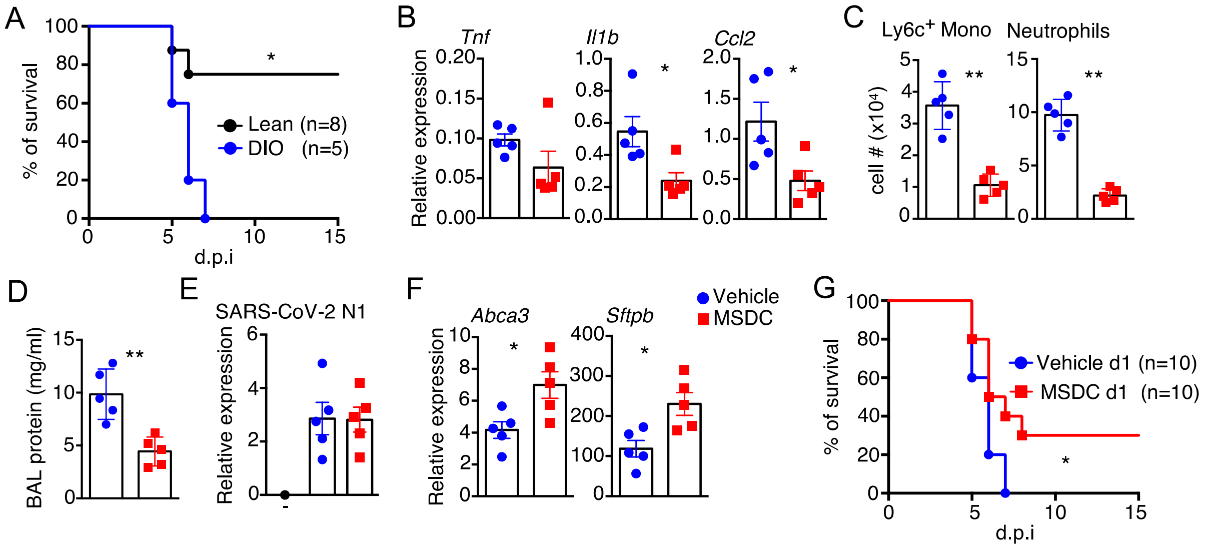


**Fig. S12. MSDC treatment decreases lung inflammation and promotes tissue recovery following SARS-CoV-2 infection in DIO mice.**

(**A**) DIO or Lean mice were infected with SARS-CoV-2 MA10 and treated with vehicle. Host mortality was monitored. (**B-F**) DIO mice were infected with SARS-CoV-2 MA10 virus and treated with vehicle or MSDC starting at 6 h post infection (n = 5). (B) The mRNA levels of *Tnf*, *Il1b* and *Ccl2* in lungs at 5 d.p.i. (C) Quantification of $Ly6c^{+}$ inflammatory monocytes ($CD11b^{+}Ly6c^{+}$) and neutrophils ($CD11b^{+}Ly6G^{+}$) in the BAL at 5 d.p.i. (D) Protein concentrations in the BAL at 5 d.p.i. (E) RT-qPCR detection of SARS-CoV-2 N1 gene in the lungs. -, negative control of IAV-infected lungs. (F) *Abca3* and *Sftpb* gene expression in the lungs at 5 d.p.i. (**G**) DIO mice were infected with SARS-CoV-2 MA10 virus and treated with vehicle or MSDC starting at 1 day post infection. Host mortality was monitored. Data are presented as means ± SEM. *, $p < 0.05$; **, $p < 0.01$. The $p$ value was determined by Logrank test (A and G) or a two-tailed Student's t-test (B to F).

Figure S13

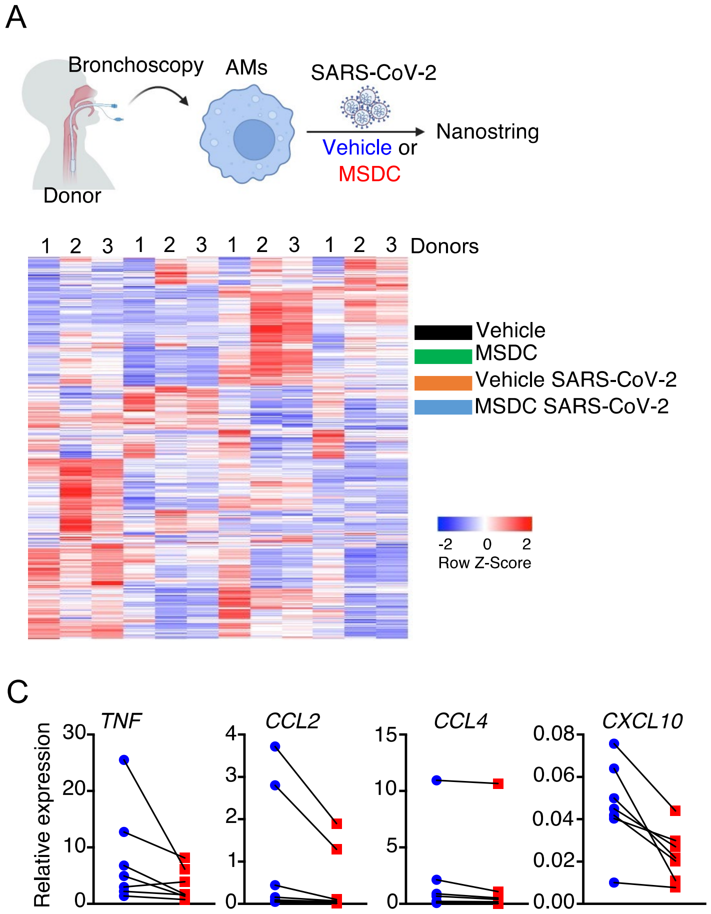


**Fig. S13. MSDC treatment reduces SARS-CoV-2-induced inflammatory cytokine expression in human AMs.**

(**A-B**) Human AMs from BAL of non-infectious donors were infected with or without SARS-CoV-2 in the presence of vehicle or MSDC (n = 3 donors) for 48 h *in vitro*. (A) Nanostring analysis of the expression of immune-related genes (human myeloid innate immunity panel) in AMs. Heatmap for total differentially expressed genes was shown. (B) Pro-inflammatory cytokine expression in AMs was measured by RT-qPCR. (**C**) Cytokine expression in lung cells from COVID-19 patient autopsies following vehicle or MSDC treatment *ex vivo* (n = 7 subjects). Target gene expression relative to *Hprt* was shown. Data are presented as means ± SEM. *, $p < 0.05$. The $p$ value was determined by one-way ANOVA (B).

Figure S14

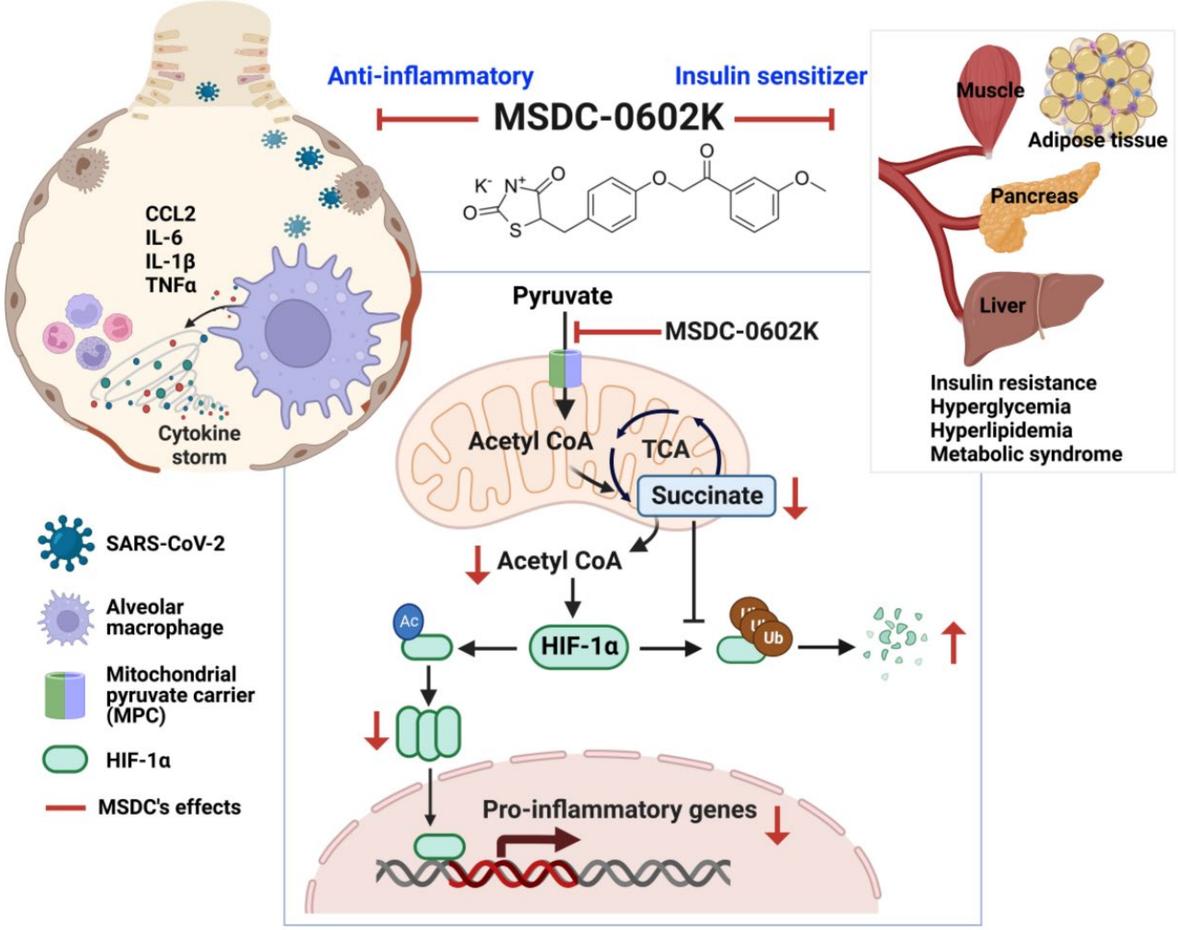


**Fig. S14. Graphical summary.**

Concurrent promotion of host metabolic health and suppression of hyper pulmonary inflammation by targeting mitochondria pyruvate translocation.

**Table S1. Primers used in quantitaive RT-PCR**

| Primers for mouse mRNA expression | | |
|---|---|---|
| Gene symbol | 5' primer (5'-3') | 3' primer (5'-3') |
| *Abca3* | TTCTGGTTCTCCGCTCTGTT | GTACATGAGGGGGATGATGG |
| *Ccl2* | GTCACCAAGCTCAAGAGAGAGGTC | CCTACAGAAGTGCTTGAGGTGGTT |
| *Hif1a* | TCAAGTCAGCAACGTGGAAG | TATCGAGGCTGTGTCGACTG |
| *Hprt* | CTCCGCCGGCTTCCTCCTCA | ACCTGGTTCATCATCGCTAATC |
| *Il1b* | GGGCCTCAAAGGAAAGAATC | TACCAGTTGGGGAACTCTGC |
| *Sftpb* | CTGTGCCAAGAGTGTGAGGA | TTGGGGTTAATCTGGCTCTG |
| *Tnf* | CATGCGTCCAGCTGACTAAA | TCCCCTTCATCTTCCTCCTT |
| | | |
| Primers for human mRNA expression | | |
| Gene symbol | 5' primer (5'-3') | 3' primer (5'-3') |
| *CCL2* | AGCAAGTGTCCCAAAGAAGC | TGGAATCCTGAACCCACTTC |
| *CCL4* | CTTCCTCGCAACTTTGTGGT | TCACTGGGATCAGCACAGAC |
| *CXCL10* | GAATCGAAGGCCATCAAGAA | CCTCTGTGTGGTCCATCCTT |
| *HPRT* | GACCAGTCAACAGGGGACAT | CTGCATTGTTTTGCCAGTGT |
| *IL1B* | GGACAAGCTGAGGAAGATGC | TCGTTATCCCATGTGTCGAA |
| *TNF* | AACCTCCTCTCTGCCATCAA | GGAAGACCCCTCCCAGATAG |
| | | |
| Primers for SARS-CoV-2 N1 expression | | |
| Gene symbol | 5' primer (5'-3') | 3' primer (5'-3') |
| *N1* | GACCCCAAAATCAGCGAAAT | TCTGGTTACTGCCAGTTGAATCTG |
